# Publication bias impacts on effect size, statistical power, and magnitude (Type M) and sign (Type S) errors in ecology and evolutionary biology

**DOI:** 10.1186/s12915-022-01485-y

**Published:** 2023-04-03

**Authors:** Yefeng Yang, Alfredo Sánchez-Tójar, Rose E. O’Dea, Daniel W. A. Noble, Julia Koricheva, Michael D. Jennions, Timothy H. Parker, Malgorzata Lagisz, Shinichi Nakagawa

**Affiliations:** 1 Evolution & Ecology Research Centre and School of Biological, Earth and Environmental Sciences, University of New South Wales, Sydney, NSW 2052 Australia; 2 Department of Biosystems Engineering, Zhejiang University, Hangzhou, 310058 China; 3 Department of Evolutionary Biology, Bielefeld University, 33615 Bielefeld, Germany; 4 School of Ecosystem and Forest Sciences, University of Melbourne, Parkville, Australia; 5 Division of Ecology and Evolution, Research School of Biology, The Australian National University, Canberra, ACT Australia; 6 Department of Biological Sciences, Royal Holloway University of London, Egham, Surrey, TW20 0EX UK; 7 Department of Biology, Whitman College, Walla Walla, WA 99362 USA

**Keywords:** Open science, Replicability, Reproducibility, Transparency, Selective reporting, Questionable research practices, *P*-hacking, Registered report, Many labs, Generalizability, Meta-research

## Abstract

Collaborative efforts to directly replicate empirical studies in the medical and social sciences have revealed alarmingly low rates of replicability, a phenomenon dubbed the ‘replication crisis’. Poor replicability has spurred cultural changes targeted at improving reliability in these disciplines. Given the absence of equivalent replication projects in ecology and evolutionary biology, two inter-related indicators offer the opportunity to retrospectively assess replicability: publication bias and statistical power. This registered report assesses the prevalence and severity of small-study (i.e., smaller studies reporting larger effect sizes) and decline effects (i.e., effect sizes decreasing over time) across ecology and evolutionary biology using 87 meta-analyses comprising 4,250 primary studies and 17,638 effect sizes. Further, we estimate how publication bias might distort the estimation of effect sizes, statistical power, and errors in magnitude (Type M or exaggeration ratio) and sign (Type S). We show strong evidence for the pervasiveness of both small-study and decline effects in ecology and evolution. There was widespread prevalence of publication bias that resulted in meta-analytic means being over-estimated by (at least) 0.12 standard deviations. The prevalence of publication bias distorted confidence in meta-analytic results, with 66% of initially statistically significant meta-analytic means becoming non-significant after correcting for publication bias. Ecological and evolutionary studies consistently had low statistical power (15%) with a 4-fold exaggeration of effects on average (Type M error rates = 4.4). Notably, publication bias reduced power from 23% to 15% and increased type M error rates from 2.7 to 4.4 because it creates a non-random sample of effect size evidence. The sign errors of effect sizes (Type S error) increased from 5% to 8% because of publication bias. Our research provides clear evidence that many published ecological and evolutionary findings are inflated. Our results highlight the importance of designing high-power empirical studies (e.g., via collaborative team science), promoting and encouraging replication studies, testing and correcting for publication bias in meta-analyses, and adopting open and transparent research practices, such as (pre)registration, data- and code-sharing, and transparent reporting.

**Supplementary Information:**

The online version contains supplementary material available at 10.1186/s12915-022-01485-y.

## Introduction

Replicable prior findings are the foundation of cumulative scientific research. However, large-scale collaborative attempts to repeat studies have demonstrated that prior findings often fail to replicate in the medical and social sciences [1–3]. This raises concerns about the reliability of previously published studies (often referred to as the ‘replication crisis’ [4]). A similar issue of low replicability is likely to occur in ecology and evolutionary biology [5] (see also [6]). Yet, systematic assessments of replicability in this field are exceedingly rare [6, 7] perhaps because of the absence of strong incentives towards conducting replication studies [7, 8], and for logistical reasons (e.g. difficulties of conducting studies of rare species or remote ecosystems [9, 10]).

There are, however, two inter-related indicators that can be used to retrospectively gauge replicability in ecology and evolutionary biology: publication bias and statistical power. Publication bias and low statistical power increase the occurrence of unreliable effect size estimates that cannot be replicated. Publication bias commonly occurs when studies with statistically significant results are published more frequently than those with statistically non-significant findings (also referred to as ‘file-drawer problem’ [11]) or are published more quickly [12, 13]. More rapid publication of statistically significant results can also lead to a decline in reported effects over time (‘decline effect’ [12, 13]). When statistically significant effects are preferentially published, smaller studies will tend to report larger effect sizes (known as ‘small-study effects’ [14]). Statistical power, by definition, is the likelihood of identifying a ‘true effect’ when it is present. It is often used as a proxy of ‘replicability probability’ (but see [15]), as studies with high statistical power are more likely to yield findings that can be replicated by other researchers compared to studies with low statistical power [16–18].

Several meta-research studies in ecology and evolutionary biology have investigated the prevalence of publication biases and low statistical power. Jennions and Moller [12] reported a statistically significant decline effect in a survey of 44 ecology and evolutionary biology meta-analyses that had been published in 2002. Using 52 meta-analyses published before 2000, Barto and Rillig [19] reached a similar conclusion. In a cumulative meta-analysis, Crystal-Ornelas and Lockwood [20] also identified a statistically significant decline in the magnitude of the effect of invasive species on species richness, using 240 papers published between 1999 and 2016. In their work, this decline effect was present consistently regardless of taxonomic groups, invasion time, or journal quality. Twenty years ago, statistical power in 10 ecology, evolution, and behaviour journals was estimated at 13–16% for small effects and 40–47% for medium effects (where small effects are *r* = 0.1 and medium effects are *r* = 0.3; *sensu* Cohen [21]) [22]. Even lower statistical power was estimated for the journal *Animal Behaviour* in 1996, 2003, and 2009 (7–8% and 23–26% to detect Cohen’s small and medium effect sizes, respectively [23]).

**Fig. 1.**
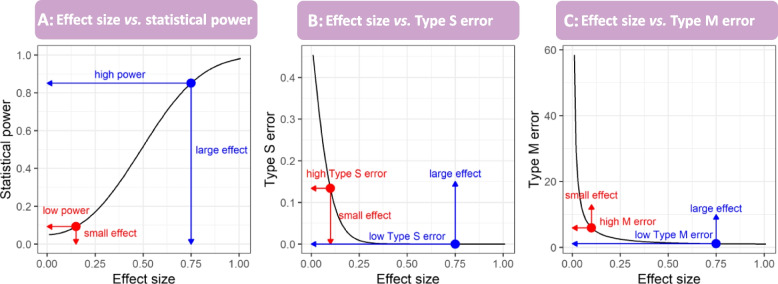
Statistical power, Type S and M errors as a function of the ‘true’ effect size (the alpha level is fixed at 0.05). The generic form of effect sizes (e.g. SMD, lnRR, *Zr*) are simulated from 0 to 1 with a fixed standard error (0.25). These panels (**A**–**C**) show that studies investigating larger true effects have higher power (**A**) and lower rates of Type M (**B**) and Type S (**C**) errors. If a study suffers from publication bias, the effect size is likely to be exaggerated, and consequently, the corresponding statistical power, Type M and S errors would be underestimated

Despite earlier efforts in ecology and evolutionary biology [24], the field still lacks a systematic overview of the extent to which different forms of publication bias would distort the estimation of true effects. Further, no studies have evaluated how such distorted effect sizes prevent us from correctly estimating statistical power. The statistical power of a given study depends on sample size and the estimate of corresponding ‘true’ effect size (e.g. a larger effect size leads to a higher power; see Fig. 1A). Therefore, to avoid overestimating the statistical power of a given study, an unbiased proxy of the ‘true’ effect size should be used. Contrastingly, previous attempts in ecology and evolution often used Cohen’s benchmarks to quantify statistical power for a given study [22, 23]. Yet, these benchmarks were derived from Cohen’s qualitative intuitions for studies in the social sciences rather than a quantitative synthesis of the representative literature [25]. Cohen’s benchmarks are arbitrary, and not necessarily applicable to ecological and evolutionary studies. As with exemplar studies in other fields [16], ‘true’ effects can be estimated via meta-analytic approaches and preferably corrected for potential publication bias [26, 27]. Using publication bias-corrected effect size estimates as ‘true’ effects would, more accurately, quantify statistical power as well as the two related, yet underappreciated, statistical errors: Type M and S errors (Fig. 1B and C; [28]). Type M error, also known as exaggeration ratio (magnitude error), represents the ratio between an estimated effect and a ‘true’ effect, whereas Type S error represents the probability of attaining statistical significance in the direction opposite to the true effect [29]. No study has yet quantified these two quantities systematically across the field of ecology and evolutionary biology.

Here, we capitalise on the rapid growth of ecological and evolutionary meta-analyses to systematically assess the extent to which patterns consistent with publication biases are common across the fields of ecology and evolutionary biology, and, if attributed to actual publication bias, their impacts on estimates of effect size, statistical power, and Type M and S errors [30]. First, we test for the presence and severity of two indices of publication bias (i.e. small-study effects and decline effects) at two levels: (i) the within-meta-analysis level using a newly proposed multilevel meta-regression method and (ii) the between-meta-analysis level using second-order meta-analyses (i.e. meta-meta-analyses). Second, we correct for these publication biases and quantify the degree of decline in bias-corrected effect-size magnitude. Finally, we use uncorrected and bias-corrected mean effect sizes as proxies of the ‘true’ effect to assess statistical power, Type M and S errors in ecology and evolutionary biology both at the primary study (effect-size) and the synthesis (meta-analysis) level.

## Methods

Before submission of stage 1 of this registered report, we finished collection (‘Data collection’ section), retrieval, and cleaning (‘Data retrieval and cleaning’ section) of data from a pre-existing dataset [31]. After this stage 1 registered report was accepted, we commenced the statistical analysis process (‘Statistical analysis’ section).

### Database

#### Data retrieval and cleaning

By checking the main text, supplementary materials, and/or online data repositories (e.g. Dryad, GitHub, Open Science Framework) of the 102 meta-analytic papers, and emailing corresponding authors, if necessary, we were able to include 80 papers that reported essential information for our statistical analyses. These 80 papers contained 108 independent meta-analyses. Among these 107, 36 meta-analyses used standardised mean difference (SMD) which includes some well-known estimators such as Hedges’ *g* or Cohen’s *d* [32]; 20 of these meta-analyses provided raw data (i.e. descriptive statistics: mean, standard error or deviation, and sample size) whereas the remaining 16 cases provided only effect sizes and variance. Twenty meta-analyses used the log response ratio (lnRR [33]; also known as the ratio of means, ROM): 10 cases with raw data, and 10 cases without raw data. Thirty-one cases used the correlation coefficient or its Fisher’s transformation, *Zr* (given that the variance of *Zr* and sample size is convertible, all cases of *Zr* were with raw data). All correlation coefficients were converted to *Zr* to better approximate normal errors [34]. The remaining 20 meta-analyses used other effect size metrics, such as heritability (*h*^2^ [35]), regression slope (e.g. reaction norm or selection gradient [36, 37]), 2-by-2 binary data (e.g. log odds and risk ratios [38]), raw mean difference [39], and non-standard metrics (proportion [40]).

**Fig. 2.**
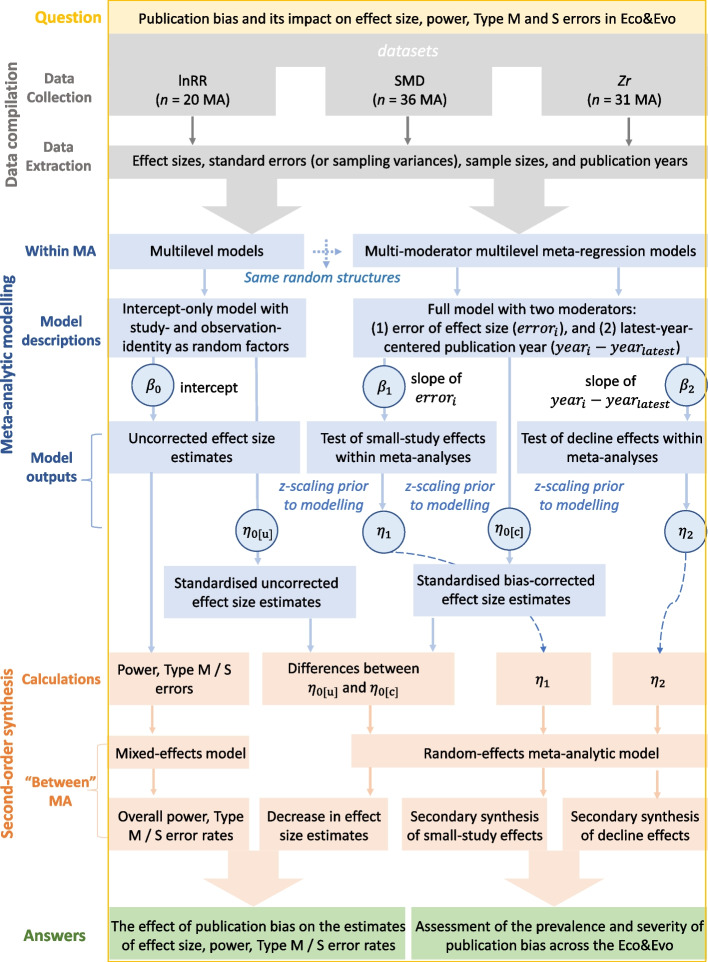
The workflow showing the data compilation, statistical modelling processes, and our aims. Using the datasets containing 87 independent meta-analyses (36 SMD, 20 lnRR and 31 *Zr* cases, respectively), we used a two-step modelling procedure to assess (i) the estimated prevalence and severity of publication bias in ecology and evolutionary biology and (ii) how such publication bias affects the estimates of effect size, statistical power, Type M and S errors. In the first step (i.e. within-meta-analysis level), multilevel meta-analytic approaches will be used to estimate the overall mean (used for power and errors calculations), and test and adjust for publication bias for each meta-analytic case. In the second step (i.e. between-meta-analysis level), the estimates from the first step were statistically aggregated using either mixed-effects models or random-effects meta-analytic models (i.e. secondary meta-analysis). *β*_0_ is the meta-analytic overall mean (i.e. *β*_0[overall]_ in Equation 1), which signifies the uncorrected effect size estimate if publication bias exists but is not corrected. *β*_1_ and *β*_2_ are the indicators of small-study effects and decline effects (equivalent to *β*_1[small − study]_ and *β*_1[time − lag]_ in Equation 2). *η*_0[u]_ is the standardised *β*_0_. (i.e. *η*_0[overall]_). *η*_0[c]_ is the standardised bias-corrected meta-analytic overall mean (i.e. *η*_0[bias − corrected]_ in Equation 6). *η*_1[small − effect]_, *η*_2[time − lag]_ are standardised model coefficients corresponding to *β*_0_, *β*_1_ and *β*_2_ (i.e. *η*_1[small − effect]_ and *η*_2[time − lag]_ in Equation 6)

We decided to only include meta-analytic cases using SMD, lnRR, and *Zr* in our datasets because, in addition to being the most commonly used effect sizes in ecology and evolutionary biology [41, 42], they share statistical properties necessary to fit a formal meta-analytic model: (i) they are ‘unit-less’, which allows comparisons of studies originally using different units, (ii) they are (asymptotically) normally distributed, and (iii) they have readily computable (unbiased) sampling variance [34]. To keep our datasets independent, we only used the effect sizes in their original forms, although data augmentations (e.g. conversions between *Zr* to SMD) could maximise the statistical power of the following statistical analyses by maximising the number of sample sizes per dataset (in this case, the number of effect sizes). Therefore, our final three datasets consisted of (1) 36 meta-analytic cases of SMD, (2) 20 cases of lnRR, and (3) 31 cases of *Zr* (Fig. 2). For each primary study included in the final dataset, we retrieved four key variables: (i) effect sizes reported (i.e. SMD, lnRR, or *Zr*), (ii) standard errors (or sampling variances) of each effect size (to test for small-study effects), (iii) sample sizes per condition where possible (i.e. experimental group *versus* control group for SMD and lnRR); sample sizes are used to create a predictor to test and correct for small-study effects (i.e. ‘effective sample size’; see the ‘Second-order meta-analysis’ section for details), and (iv) publication year (to test for a decline effect).

### Statistical analysis

#### Data collection

We used a recent meta-analytic database that had been collected to evaluate the reporting quality of systematic reviews and meta-analyses published in ecology and evolutionary biology [31]. The inclusion and screening criteria identified meta-analyses that were broadly representative of meta-analyses published in ecology and evolutionary biology journals from 2010-2019. In brief, the database creators compiled a list of ‘Ecology’ and/or ‘Evolutionary Biology’ journals via the categories of the ISI InCites Journal Citation Reports®. Within the included journals, they searched Scopus using the string ‘meta-analy*’ OR ‘metaanaly*’ OR ‘meta-regression’. They restricted the search to articles published from January 2010 to 25 March 2019. Search results were then filtered to the 31 journals most frequently publishing meta-analyses. By taking a random sample of studies within each journal, a total of 297 papers was returned. After screening (search records, and inclusion and screening criteria are available at [31]), the database included a representative sample of 102 ecological or evolutionary meta-analyses.

#### Multilevel meta-analytic modelling

We used multilevel meta-analytic approaches to (i) estimate the meta-analytic overall mean (i.e. uncorrected effect size estimates), (ii) detect potential publication bias (i.e. test small-study and decline effects), and (iii) correct for publication bias for each meta-analysis included in our datasets (Fig. 2).

##### Estimating uncorrected effect sizes

To obtain uncorrected effect sizes for each meta-analysis (i.e. within-meta-analysis level), we fitted intercept-only multilevel meta-analytic models with SMD, lnRR, and *Zr* as our response variables, as in Equation 1 [42]. Equation 1 can account for dependent data by modelling both between-study variance (heterogeneity) and within-study variance (residual). It was written as:

1 $${ES}_{ji}={\beta}_{\textrm{0}\left[\textrm{overall}\right]}+{s}_j+{o}_{ji}+{m}_{ji},$$

where *ES*_*ji*_ is the extracted effect size, either SMD, lnRR, or *Zr*; *β*_0[overall]_ is the intercept, representing the estimate of overall effect (i.e. meta-analytic estimate of effect size); *s*_*j*_ = the study-specific (between-study) effect of study *j*; *o*_*ji*_ = the observation-level (within-study) effect for the effect size *i* (used to account for residual heterogeneity); *m*_*ji*_ = the measurement (sampling) error effect for the effect size *i*. Between- and within-study effects are normally distributed with mean 0 and variance, *σ*^2^ (i.e. $\mathcal{N}\left(0,{\sigma}^2\right)$). In Equation 1, effect size (*ES*_*ji*_) and sampling variance (*m*_*ji*_) can be calculated from the meta-analytic data. Using the restricted maximum likelihood (REML) method, we can obtain (approximately) unbiased estimates of variance parameters *σ*^2^ for between- and within-study effects (*s*_*j*_ and *o*_*ji*_) [43]. With the REML estimate of *σ*^2^, we can obtain the maximum likelihood estimate of the model coefficients (i.e. *β*_0[overall]_). These estimated model coefficients represent the (uncorrected) overall meta-analytic means for SMD, lnRR, or *Zr*. The model fitting was implemented via the (*rma.mv*) function from the *metafor* R package (version 3.4-0) [44].

##### Detecting publication bias

To test for patterns consistent with publication bias within each meta-analysis, we used a multi-moderator multilevel meta-regression model (an extended Egger’s regression; cf. [45]). This approach deals with two common issues in ecological and evolutionary datasets: (i) using a multilevel model to control for data dependency [46], and (ii) using a regression method with multiple moderators to account for between-study heterogeneity [47]. We adopted this approach to test the presence of small-study and decline effects, respectively. This was written as:

2 $${ES}_{ji}={\beta}_{0\left[\textrm{bias}-\textrm{corrected}\right]}+{\beta}_{1\left[\textrm{small}-\textrm{study}\right]}{error}_i+{\beta}_{2\left[\textrm{time}-\textrm{lag}\right]}\left({year}_i-{year}_{latest}\right)+{s}_j+{o}_{ji}+{m}_{ji},$$

where *β*_0[bias − corrected]_ is the intercept, representing bias-corrected overall effect/meta-analytic estimate of effect size (see more details below); *error*_*i*_ is the uncertainty index of effect size (i.e. sampling error of effect size, *se*_*i*_), and *β*_1[small − study]_ is the corresponding slope and an indicator of small-study effects; *year*_*i*_ is the publication year, *year*_*latest*_ is the latest year of published papers, and *β*_2[time − lag]_ is the corresponding slope and an indicator of decline effect (i.e. time-lag bias).

When assuming there is no small-study effect (i.e. *error*_*i*_ = 0) and decline effect (i.e. *year*_*i*_ − *year*_*latest*_ = 0), the intercept *β*_0[bias-corrected]_ in Equation 2 becomes a conditional estimate that can be interpreted as the bias-corrected overall effect (i.e. the estimate of ‘true’ effect which is distinct from the unconditional estimate of *β*_0[overall]_ in Equation 1). We centred the ‘year’ variable by subtracting each year (*year*_*i*_ ) from the latest *year*_*latest*_ to set the latest year as the intercept, *β*_0[bias − corrected]_. This process allowed the estimate of true effect (i.e. *β*_0[bias − corrected]_ in Equation 2) to be conditional on *year*_*i*_ = *year*_*latest*_ so that *β*_0_ was least affected by a decline effect if it existed. Further, we used a sampling error equivalent $\sqrt{1/\tilde{n}_i}=\sqrt{\left({n}_e+{n}_c\right)/{n}_e{n}_c}$) to replace *se*_*i*_ when fitting SMD and lnRR where possible ($4\tilde{n}_i$ is referred to as an effective sample; *n*_*e*_ is the sample size of the experimental group, *n*_*c*_ is the sample size of the control group [45]). This can correct for the ‘artefactual’ correlation between *ES*_*ji*_ and *error*_*i*_ as the point estimate of SMD and lnRR are inherently correlated with their sampling variances (see Table 3 in [34], and Equation 10 in [48]).

A small-study effect is statistically detected if Equation 2 has a statistically significant *β*_1[small − study]_ (i.e. *p*-value < 0.05). Similarly, the decline effect (i.e. time-lag bias) is indicated by a statistically significant *β*_2[time − lag]_. Depending on the specific phenomenon tested, *β*_1[small − study]_ and *β*_2[time − lag]_ might be expected to be positive or negative when publication bias exists. For example, for an effect that is expected to be positive, a small-study effect and decline effect would be expressed in a positive value of *β*_1[small − study]_ (i.e. small-size non-statistically significant effects and small-size statistically significant negative effects are underrepresented)) and negative value of *β*_2[time − lag]_ (i.e. overall effect size declines over time), respectively. In such a case, a slope (*β*_1[small − study]_ or *β*_2[time − lag]_) with opposing direction (unexpected sign) indicates no detectable publication bias and subsequently does not require correction for such a bias. The magnitude of the slope represents the severity of the small-study effect or decline effect. Therefore, using Equation 2, we were able to detect the existence of publication bias and identify its severity for each meta-analysis and each effect size statistic.

##### Correcting overall estimates for publication bias

To avoid the biased estimate of *β*_0[bias − corrected]_, we fitted Equation 3 when detecting a statistically significant *β*_0[bias − corrected]_ in Equation 2. Equation 3 was written as:

3 $${ES}_{ji}={\beta}_{0\left[\textrm{bias}-\textrm{corrected}\right]}+{\beta}_{1\left[\textrm{small}-\textrm{study}\right]}{error}_i^2+{\beta}_{2\left[\textrm{time}-\textrm{lag}\right]}\left({year}_i-{year}_{latest}\right)+{s}_j+{o}_{ji}+{m}_{ji},$$

In contrast to Equation 2, Equation 3 used a quadratic term of uncertainty index (i.e. sampling variance *v*_*i*_ or $1/\tilde{n}_i$) to alleviate the downward bias of an effect size estimate (for explanations see [45, 49]). Theoretically, this procedure provided an easy-to-implement method to correct for publication bias for each meta-analysis (i.e. the conditional estimate of intercept in Equation 3). In practice, however, there were two different types of *β*_0[bias − corrected]_ estimates to consider. This is because high heterogeneity [47] can lead the signs of the slopes (*β*_1[small − study]_ and *β*_2[time − lag]_) to be opposite from that expected from publication bias [45]. We would subsequently misestimate *β*_0[bias − corrected]_ if slopes with unexpected signs are included in Equations 2 and 3.

**Fig. 3.**
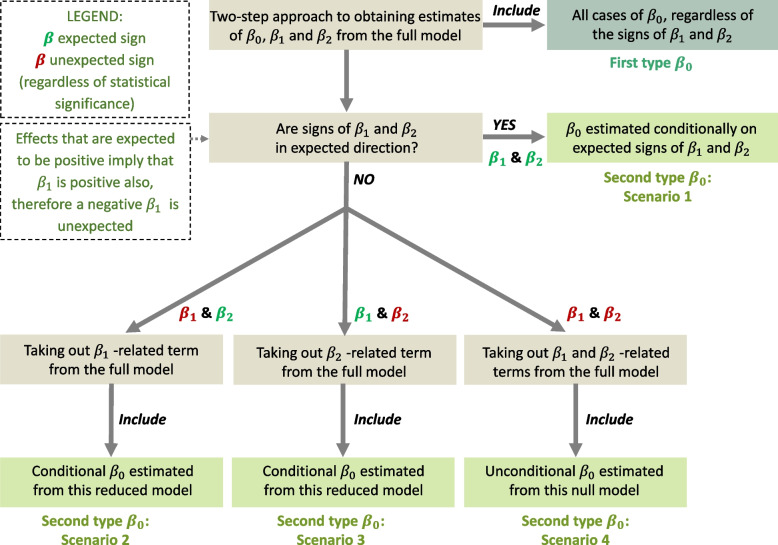
The decision tree used to obtain the estimate of the ‘unbiased’ effect (i.e. conditional *β*_0_). First, use a two-step procedure to estimate *β*_0_, *β*_1_ and *β*_2_ from the full model (Equations 2 or 3). Then, depending on whether the signs of slopes (*β*_1_ and *β*_2_) are opposite from what will be expected from publication bias (caused by a high amount of unaccounted heterogeneity), there are two types of estimates of *β*_0_. The first type includes all *β*_0_ regardless of their signs (*β*_1_ and *β*_2_); the second type of estimated *β*_0_ has four scenarios. Scenario 1 = only select *β*_0_ with expected signs of *β*_1_ and *β*_2_ from the full model; Scenario 2 = employ reduced model 1 (Equation 4) to re-estimate *β*_0_ where *β*_1_ has an unexpected sign, while *β*_2_ has an expected sign; Scenario 3 = employ reduced model 3 (Equation 5) to re-estimate *β*_0_ if *β*_1_ has an expected sign, while *β*_2_ has an unexpected sign; Scenario 4 = use *β*_0_ from the null model (Equation 1) when both *β*_1_ and *β*_2_ have unexpected signs (i.e. without the small-study effects or decline effects). The symbols (*β*_0_, *β*_1_, and *β*_2_) are as in Fig. 2

Depending on the signs of the slopes (*β*_1[small − study]_ and *β*_2[time − lag]_), there were two types of estimated *β*_0[bias − corrected]_. We used a decision tree (Fig. 3) to obtain the estimate of each type of *β*_0[bias − corrected]_ for each meta-analytic case. The function of the decision tree was that, if the slopes (*β*_1[small − study]_ and *β*_2[time − lag]_) had unexpected signs, we took out the corresponding slope-related term(s) from the full models to form reduced models (Equations 4 and 5) to better estimate *β*_0_. The reduced models were written as Equations 4 and 5, respectively:

4 $${ES}_{ji}={\beta}_{0\left[\textrm{bias}-\textrm{corrected}\right]}+{\beta}_{1\left[\textrm{small}-\textrm{study}\right]}{error}_i+{s}_j+{o}_{ji}+{m}_{ji},$$

5 $${ES}_{ji}={\beta}_{0\left[\textrm{bias}-\textrm{corrected}\right]}+{\beta}_{2\left[\textrm{time}-\textrm{lag}\right]}\left({year}_i-{year}_{latest}\right)+{s}_j+{o}_{ji}+{m}_{ji},$$

Specifically, the first type of estimate of *β*_0[bias − corrected]_ was obtained by fitting Equation 2 or 3 (termed as full models). That included all cases of *β*_0[bias − corrected]_ without consideration of the signs of *β*_1_ and *β*_2_ (i.e. conditional *β*_0[bias − corrected]_ estimated from the full model; see Fig. 3). The second type of estimate of *β*_0[bias − corrected]_ was obtained under the following four scenarios: (i) *β*_0[bias − corrected]_ estimated under expected signs of *β*_1[small − study]_ and *β*_2[time − lag]_ (i.e. conditional *β*_0[bias − corrected]_ estimated from the direction-controlled full model; see Fig. 3), which meant a co-occurrence of a small-study effect and a decline effect, (ii) *β*_0[bias − corrected]_ estimated under the expected sign of *β*_1[small − study]_ and the unexpected sign of *β*_2[time − lag]_, which signalled the existence of a small-study effect but no decline effect (i.e. conditional *β*_0[bias − corrected]_ estimated from reduced model 1; see Equation 4 and Fig. 3), (iii) *β*_0[bias − corrected]_ estimated under the unexpected sign of *β*_1_ and the expected sign of *β*_2_, which indicated the occurrence of a decline effect but no small-study effect (i.e. conditional *β*_0[bias − corrected]_ estimated from reduced model 2; see Equation 5 and Fig. 3), and (iv) *β*_0[bias − corrected]_ estimated under unexpected signs of *β*_1[small − study]_ and *β*_2[time − lag]_, which suggested little concerns about a small-study effect or a decline effect.

#### Second-order meta-analysis

In this section, we statistically aggregated the above-mentioned regression coefficients (i.e. *β*_0[bias − corrected]_, *β*_1[small − study]_ and *β*_2[time − lag]_) to (i) reveal the patterns of potential publication bias across the fields of ecology and evolutionary biology, and (ii) quantify the extent to which publication bias might cause a reduction in effect-size magnitude across meta-analyses (Fig. 2).

##### Estimating the overall extent and severity of publication bias

To allow for aggregations of *β*_1[small − study]_ (i.e. an indicator of small-study effect) and *β*_2[time − lag]_ (i.e. an indicator of decline effect) over different effect size metrics (i.e. SMD, lnRR, and *Zr*), we standardised coefficients to eliminate scale-dependency [50]. This was achieved by *z*-scaling (i.e. mean-centring and dividing by the standard deviation) *error*_*i*_, *year*_*i*_ − *year*_*latest*_, and standardising the response variable *ES*_*ji*_ by dividing by the standard deviation without mean-centring, prior to modelling, as given by Equation 6:

6 $$c\left({ES}_{ji}\right)={\eta}_{0\left[\textrm{bias}-\textrm{corrected}\right]}+{\eta}_{1\left[\textrm{small}-\textrm{effect}\right]}z\left({error}_i\right)+{\eta}_{2\left[\textrm{time}-\textrm{lag}\right]}z\left({year}_i-{year}_{latest}\right)+{s}_j+{o}_{ji}+{m}_{ji},$$

Equation 6 indicates that one standard deviation change in *error*_*i*_ and *year*_*i*_ − *year*_*latest*_ would change *ES*_*ji*_ by *η*_1[small − effect]_ and *η*_2[time − lag]_ standard deviations, respectively. Further, to interpret *β*_0_ as a bias-corrected overall effect, *β*_0_ was set conditional on *error*_*i*_ = 0 (i.e. without small-study effect) and *year*_*i*_ − *year*_*latest*_ = 0 (i.e. without decline effect). As such, we replaced *z*(*error*_*i*_) by *z*(*error*_*i*_) − *z*(*error*_0_) and replace *z*(*year*_*i*_ − *year*_*latest*_) by *z*(*year*_*i*_) − *z*(*year*_*latest*_), as shown in Equation 7:

7 $$c\left({ES}_{ji}\right)={\eta}_{0\left[\textrm{bias}-\textrm{corrected}\right]}+{\eta}_{1\left[\textrm{small}-\textrm{effect}\right]}\left(z\left({error}_i\right)-z\left({error}_0\right)\right)+{\eta}_{2\left[\textrm{time}-\textrm{lag}\right]}\left(z\left({year}_i\right)-z\left({year}_{latest}\right)\right)+{s}_j+{o}_{ji}+{m}_{ji},$$

where *z*(*error*_0_) denotes the *z*-score when *error*_*i*_ = 0, which is equal to $\frac{0-\textrm{mean}\left[{error}_i\right]}{\textrm{SD}\left[{error}_i\right]}$; *z*(*year*_*latest*_) is the *z*-score when *year*_*i*_ is the latest year. Likewise, to obtain the best estimate of standardised bias-corrected effects, we introduced Equation 8 where a quadratic error term was used:

8 $$c\left({ES}_{ji}\right)={\eta}_{0\left[\textrm{bias}-\textrm{corrected}\right]}+{\eta}_{1\left[\textrm{small}-\textrm{effect}\right]}\ {\left(z\left({error}_i\right)-z\left({error}_0\right)\right)}^2+{\eta}_{2\left[\textrm{time}-\textrm{lag}\right]}\ \left(z\left({year}_i\right)-z\left({year}_{latest}\right)\right)+{s}_j+{o}_{ji}+{m}_{ji},$$

Therefore, fitting 8 created two datasets: (1) the full dataset containing *η*_0[bias − corrected]_, *η*_1[small − effect]_ and *η*_2[time − lag]_ without consideration of their signs (standardised slopes of the first type estimate), and (2) the reduced dataset containing *η*_0[bias − corrected]_, *η*_1[small − effect]_ and *η*_2[time − lag]_ with expected directions (standardised slopes of the second type estimate: scenarios 1–4, Fig. 3). We then conducted a series of second-order meta-analyses to statistically aggregate these standardised regression coefficients across meta-analyses [51, 52]. We employed a random-effects meta-analytic model with the inverse square of each coefficient’s standard error as weights to fit such second-order meta-analyses [44]. For both the full and reduced databases, we obtained a weighted average of the regression coefficient *η*_1[small − effect]_ (or *η*_2[time − lag]_) to indicate the occurrence of small-study effects (or decline effects) across the fields of ecology and evolutionary biology. To compare the severity of publication bias between different types of effect size, we further incorporated effect-size types as a moderator (i.e. a fixed factor or predictor with three levels: SMD, lnRR, and *Zr*) in these random-effects models.

##### Quantifying the reduction in effect-size magnitude after controlling for publication bias

Likewise, to quantify the differences between uncorrected effect sizes and their bias-corrected estimates for the different types of effect-size metrics, we required standardised estimates of these effect sizes to draw comparisons. The term *η*_0[bias − corrected]_ in the full dataset provided a standardised bias-corrected effect size (i.e. an intercept estimated using the full model, where all cases of *η*_1[small − effect]_ and *η*_2[time − lag]_ were included regardless of their directions). Also, *η*_0[bias − corrected]_ in the reduced dataset provided standardised bias-corrected effect sizes, which were obtained using expected directions of *η*_1[small − effect]_ and *η*_2[time − lag]_. In contrast, the standardised uncorrected effect sizes were obtained via standardising *ES*_*ji*_ by dividing by standard deviation before fitting Equation 1 (that is, standardised intercept in the null model: *η*_0[overall]_). We then used the absolute mean difference as a metric to quantify the reduction in effect-size magnitude following correction for publication bias, where the point estimate and sampling variance was written as:

9 $$D=\mid {\upgamma}_{\textrm{uncorrected}-\textrm{effect}}^s-{\upgamma}_{\textrm{corrected}-\textrm{effect}}^s\mid,$$

10 $$\textrm{Var}(D)={\textrm{SE}}_{\upgamma_{\textrm{corrected}-\textrm{effect}}^s}^2+{\textrm{SE}}_{\upgamma_{\textrm{uncorrected}-\textrm{effect}}^s}^2-2r{\textrm{SE}}_{\upgamma_{\textrm{corrected}-\textrm{effect}}^s}{\textrm{SE}}_{\upgamma_{\textrm{uncorrected}-\textrm{effect}}^s},$$

where ${\upgamma}_{\textrm{corrected}-\textrm{effect}}^s$ and ${\upgamma}_{\textrm{uncorrected}-\textrm{effect}}^s$ are the values of standardised uncorrected effect size (standardised *η*_0[overall]_ in the null model) and its bias-corrected version (standardised *η*_0[bias − corrected]_ in the full or reduced models), respectively; ${\textrm{SE}}_{\upgamma_{\textrm{corrected}-\textrm{effect}}^s}$ and ${\textrm{SE}}_{\upgamma_{\textrm{uncorrected}-\textrm{effect}}^s}$ are associated standard errors; *r* is the correlation between standard errors (${\textrm{SE}}_{\upgamma_{\textrm{corrected}-\textrm{effect}}^s}$*vs.* and ${\textrm{SE}}_{\upgamma_{\textrm{uncorrected}-\textrm{effect}}^s}$), which is assumed to be 1 because the two estimates should be strongly correlated.

Given that *D* is an absolute variable, it follows a ‘folded’ normal distribution because taking the absolute value will force probability density on its left side (*x*-axis < 0) to be folded to the right [53, 54]. The corresponding folded mean and variance could be derived from its ‘folded’ normal distribution as Equations 11 and 12:

11 $${D}_f=\sqrt{\frac{2}{\pi}\textrm{Var}(D)}{e}^{-{D}^2/2\textrm{Var}(D)}+D\left(1-2\Phi \left(\frac{-D}{\sqrt{\textrm{Var}(D)}}\right)\right),$$

12 $$\textrm{Var}\left({D}_f\right)={D}^2+\textrm{Var}\left(\textrm{D}\right)-{\left(\sqrt{\frac{2}{\pi}\textrm{Var}\left(\textrm{D}\right)}{e}^{-{D}^2/2\textrm{Var}\left(\textrm{D}\right)}+D\left(1-2\Phi \left(\frac{-D}{\sqrt{\textrm{Var}\left(\textrm{D}\right)}}\right)\right)\right)}^2,$$

where Φ is the standard normal cumulative distribution function (see more details in [53, 55]). Equations 9 to 12 enable us to calculate *D*_*f*_ and Var(*D*_*f*_) for both full and reduced databases. We used a random-effects meta-analytic model ((*rma.uni*) function [44]) to synthesise these *D*_*f*_ with Var(*D*)_*f*_ as sampling variance across meta-analyses. Also, we incorporated effect size type as a moderator to compare the differences in effect size reduction between SMD, lnRR, and *Zr*.

#### Estimating statistical power, and type M and S errors

We assessed the statistical power and Type M and S errors in the primary studies with experimental effects that were approximated by uncorrected and bias-corrected effect sizes [27, 56]. Although meta-analyses can increase power over primary studies [57], they might still be underpowered to detect the true effect (i.e. *p*-value > 0.05 despite the existence of a true effect). Therefore, we also calculated the statistical power, Type M and S errors for each meta-analysis. To obtain average statistical power, and Type M and S errors at the primary study level, we used a linear mixed-effects model to aggregate over the estimates of power, and Type M and S errors from primary studies. We used the (*lmer*) function in the *lme4* R package (version 1.1-26) to fit these mixed-effects models [58], which incorporated the identity of the primary study as a random factor to account for between-study variation. Similarly, we used a weighted regression to aggregate meta-analysis level power, and Type M and S errors, with the number of effect sizes (*k*) within each meta-analysis as weights. We implemented the weighted regression via the *base R* function (version 4.0.3), (*lm*).

#### Deviations and additions

The Stage 2 of this registered report has three deviations from the Stage 1 protocol. First, in the ‘Correcting for overall estimates for publication bias’ section, the best estimate of the bias-corrected overall effect (i.e. model intercept *β*_0[bias − corrected]_) was initially planned to be obtained by a two-step procedure where when a zero effect exists (i.e. statistically non-significant *β*_0[bias − corrected]_), uncertainty index (i.e. sampling error *error*_*i*_ or $\sqrt{1/\tilde{n}_i}$) was used (Equation 2) to estimate *β*_0[bias − corrected]_, while when a non-zero effect exists (i.e. statistically significant *β*_0[bias − corrected]_), a quadratic term of uncertainty index (i.e. sampling variance *v*_*i*_ or $1/\tilde{n}_i$) was used (Equation 3) to estimate *β*_0[bias − corrected]_ [59, 60]. We decided to only use Equation 3 to estimate *β*_0[bias − corrected]_ because there is no need to estimate *β*_0[bias − corrected]_ when no statistically significant effect exists (Equation 2).

Second, in the ‘Estimating the overall extent and severity of publication bias’ section, we changed *z*-scaling (i.e. mean-centring and dividing by the standard deviation) response variable *ES*_*ji*_ prior to model fitting to standardising response variable *ES*_*ji*_ by dividing by the standard deviation without mean-centring. This is because centring the response variable would make estimating model intercept (*β*_0[bias − corrected]_) unfeasible [50]. The same change was made in the ‘Quantifying the reduction in effect-size magnitude after controlling for publication biases’ section.

Third, we added a post hoc analysis where we removed the meta-analyses with statistically non-significant mean effects and subsequently calculated the average statistical power, Type M and S error rates. The reason why adding this post hoc analysis was that the underlying true effect sizes in some meta-analyses were likely to be so trivially small (and biologically insignificant) that corresponding power calculation was meaningless. In such a case, if we included those effects when estimating average power across meta-analyses in ecology and evolution, we would get a downwardly biased average power estimate. Note that relevant results were reported in Supplementary Material (Table S4).

## Results

### The pattern of small-study effects in ecology and evolutionary biology

#### Within-meta-analysis level

**Fig. 4.**
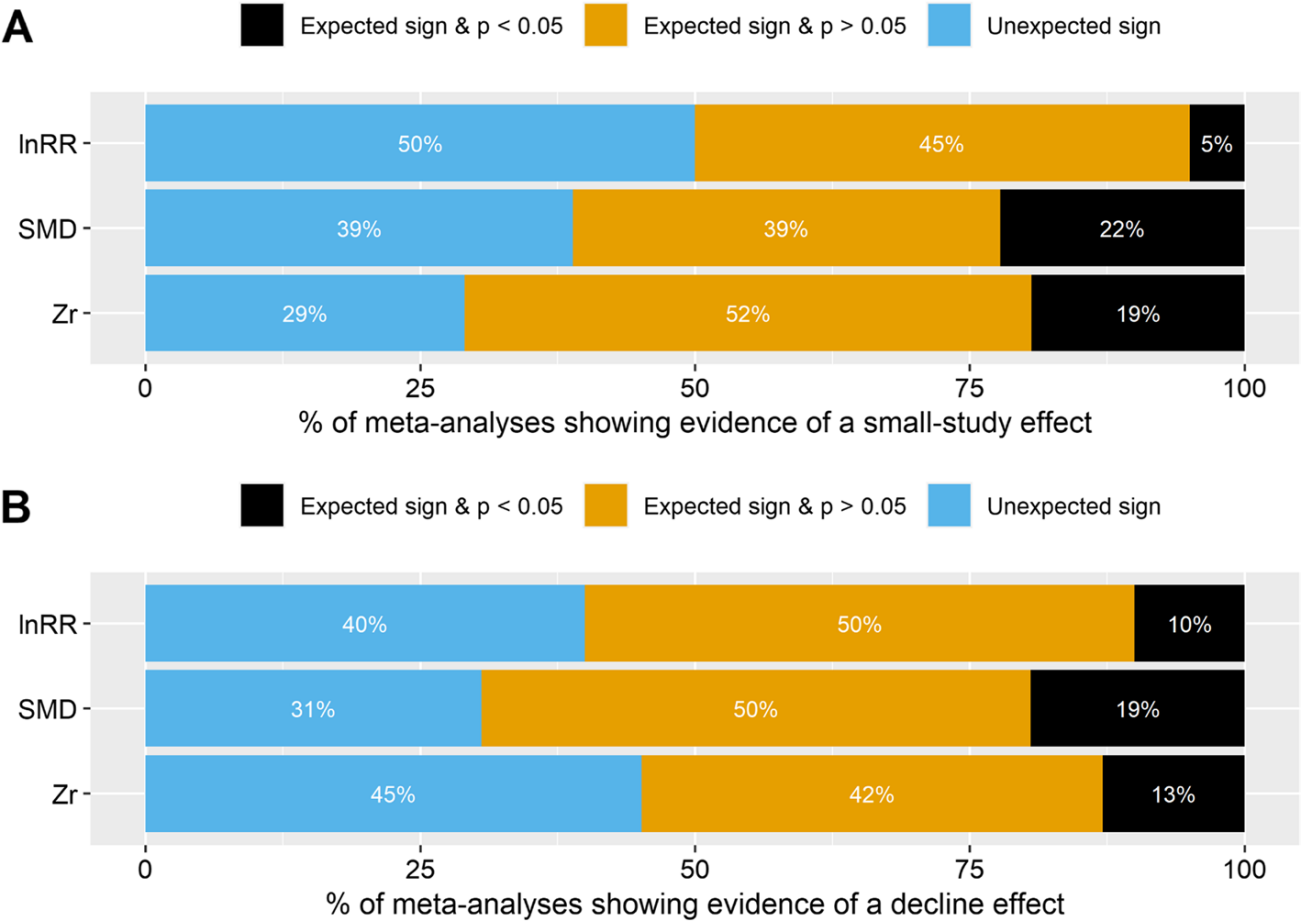
The percentage of ecology and evolutionary meta-analyses showing evidence of publication bias. **A** A small-study effect (i.e. small non-statistically significant effects and small statistically significant effects of opposite direction to the overall effect are underrepresented). **B** A decline effect (the magnitude of effect sizes changes over time). See more details in the legend of Fig. 3. All figures were drawn using the *geom_bar()* function in *ggplot2* R package (version 3.3.5) [61]

Of the 87 ecological and evolutionary meta-analyses tested, 15 (17%) meta-analyses showed evidence for small-study effects (i.e. statistically significant *β*_1[small − study]_; see Fig. 4A), where smaller studies reported larger effect sizes. Importantly, *β*_1[small − study]_ from 54 (62%) meta-analyses were in the expected direction (Fig. 4A), indicating that these meta-analyses exhibited a (statistically non-significant) tendency for a small-study effect (note that the likelihood of a meta-analysis to show this tendency is 50% if there is no real effect).

#### Between-meta-analysis level

**Fig. 5.**
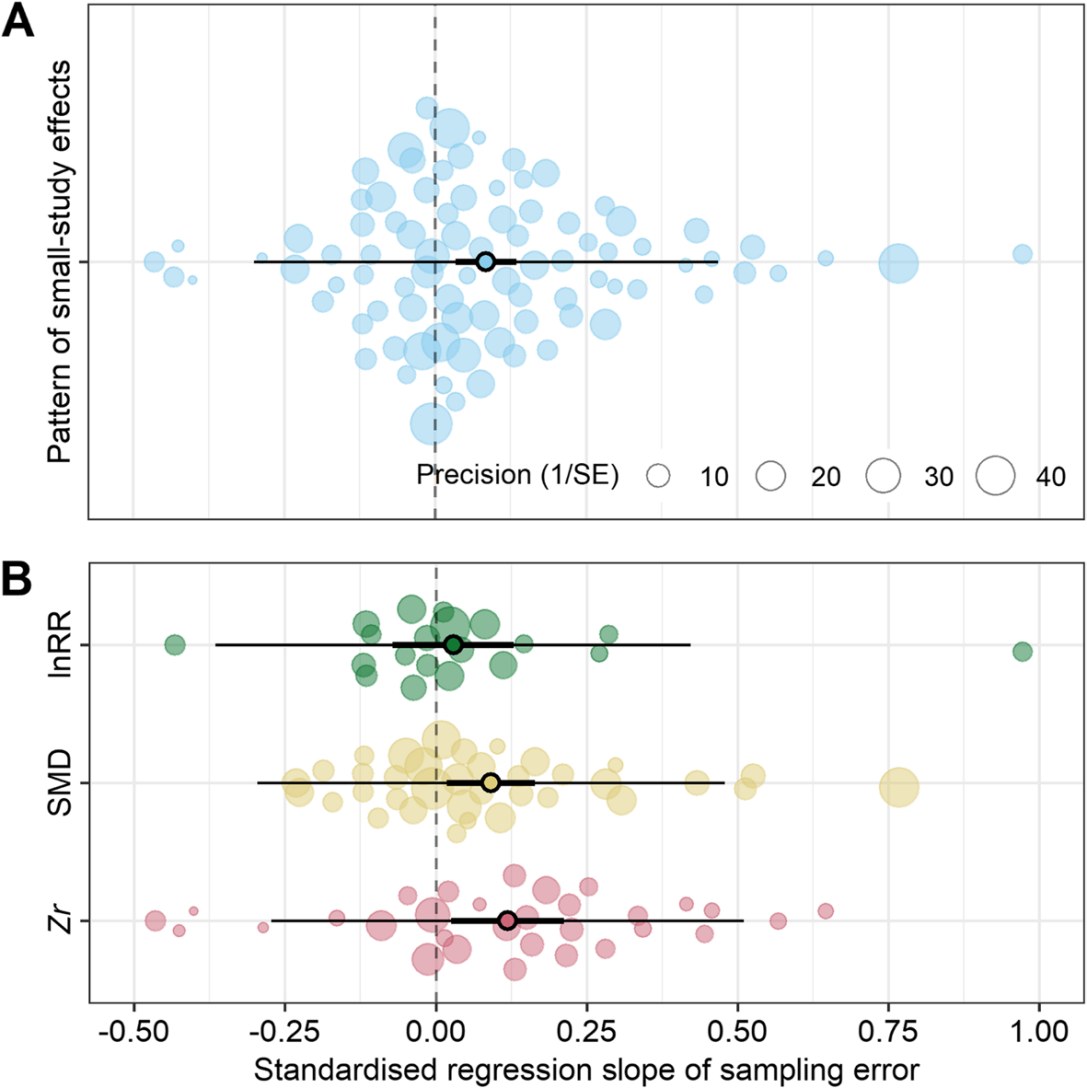
Orchard plots showing the distribution of the indicator of small-study effect (model slope *β*_1[small − study]_) for each meta-analysis and meta-analytic aggregation of *β*_1[small − study]_ (pooled *β*_1[small − study]_). (A) Pooled *β*_1[small − study]_ across different meta-analyses and different types of effect size, indicating the pattern of small-study effects. (B) Pooled *β*_1[small − study]_ for each type of effect size. Solid circles = *β*_1[small − study]_ estimates obtained from each meta-analysis; the size of each solid circle is proportional to its inverse standard error (i.e. precision). Open circles = pooled *β*_1[small − study]_. Thick error bars = 95% confidence intervals (CI). Thin error bars = prediction intervals (PIs). See more details in the legend of Fig. 2. All panels were made using *orchard_plot()* function in *orchaRd* R package (version 2.0) [62]

When conducting a second-order meta-analysis by aggregating the *β*_1[small − study]_ obtained from the 87 meta-analyses, there was a statistically significant pooled *β*_1[small − study]_ (grand mean *β*_1[small − study]_ = 0.084, 95% confidence intervals (CI) = 0.034 to 0.135, *p*-value = 0.001, *N* = 87; Fig. 5A). This provides statistical evidence for the existence of small-study effects across the meta-analyses. Furthermore, the heterogeneity among the *β*_1[small − study]_ estimates obtained from the 87 meta-analyses was low (${\sigma}_{among- meta- analysis}^2$ = 0.0050; ${I}_{among- meta- analysis}^2$ = 10%), indicating that these results are highly generalizable. Three per cent of this heterogeneity could be explained by the types of effect sizes (SMD, lnRR, *Zr*) being meta-analyzed (${R}_{marginal}^2$ = 0.031). The non-random pattern of the small-study effect was mainly driven by SMD (grand mean *β*_1[small − study]_ = 0.091, 95% CI = 0.018 to 0.165, *p*-value = 0.015, *N* = 36) and *Zr* (grand mean *β*_1[small − study]_ = 0.119, 95% CI = 0.026 to 0.212, *p*-value = 0.013, *N* = 20), but not lnRR (grand mean *β*_1[small − study]_ = 0.029, 95% CI = −0.072 to 0.130, *p*-value = 0.571, *N* = 31).

### The pattern of decline effects in ecology and evolutionary biology

#### Within-meta-analysis level

Out of the 87 ecological and evolutionary meta-analyses reviewed, 13 (15%) revealed evidence of a decline effect, where the effect sizes significantly decreased over time (Fig. 4B). Additionally, 54 (62%) of the meta-analyses showed a statistically non-significant decline in effect size over time.

#### Between-meta-analysis level

**Fig. 6.**
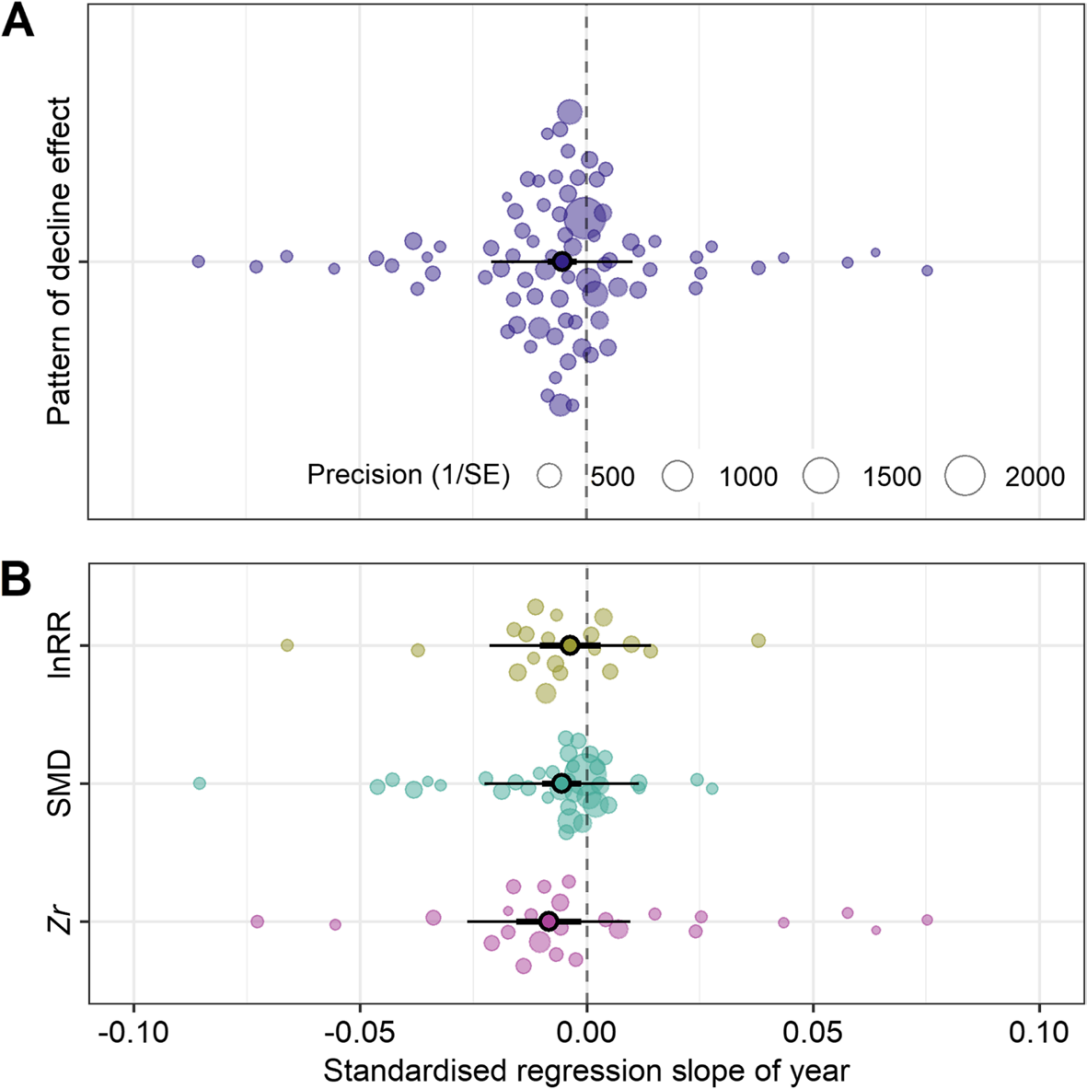
Orchard plots showing the distribution of the indicator of decline effects (model slope *β*_2[time − lag]_) for each meta-analysis and meta-analytic aggregation of *β*_2[time − lag]_ (pooled *β*_2[time − lag]_). **A** Pooled *β*_2[time − lag]_ across different meta-analyses and different types of effect size, indicating the systematic pattern of decline effect. **B** Pooled *β*_2[time − lag]_ for each type of effect size. See more details in the legend of Figs. 2 and 3. All panels were made using *orchard_plot()* function in *orchaRd* R package (version 2.0) [62]

There was a statistically significant pooled *β*_2[time − lag]_ (grand mean *β*_2[time − lag]_ = −0.006, 95% CI = −0.009 to −0.002, *p*-value < 0.001; Fig. 6A) across 87 meta-analyses, providing statistical evidence for the existence of decline effects. The estimates of *β*_2[time − lag]_ were homogeneous across these meta-analyses, indicating high generalizability of the results, with a low relative heterogeneity (${\sigma}_{among- meta- analysis}^2$ = 0.0001; ${I}_{among- meta- analysis}^2$ < 1%). Five per cent of that heterogeneity could be explained by the types of effect sizes (${R}_{marginal}^2$ = 0.05); SMD and *Zr* exhibited a statistically significant pattern of decline effect (SMD: pooled *β*_2[time − lag]_ = −0.005, 95% CI = −0.010 to −0.001, *p*-value = 0.013, *N* = 36; *Zr*: pooled *β*_2[time − bias]_ = −0.008, 95% CI = −0.015 to −0.001, *p*-value = 0.023, *N* = 31; Fig. 6B), but lnRR did not (pooled *β*_2[time − bias]_ = −0.004, 95% CI = −0.010 to 0.003, *p*-value = 0.289, *N* = 20).

### The inflation of effect size estimates and distortion of meta-analytic evidence by publication bias

**Fig. 7.**
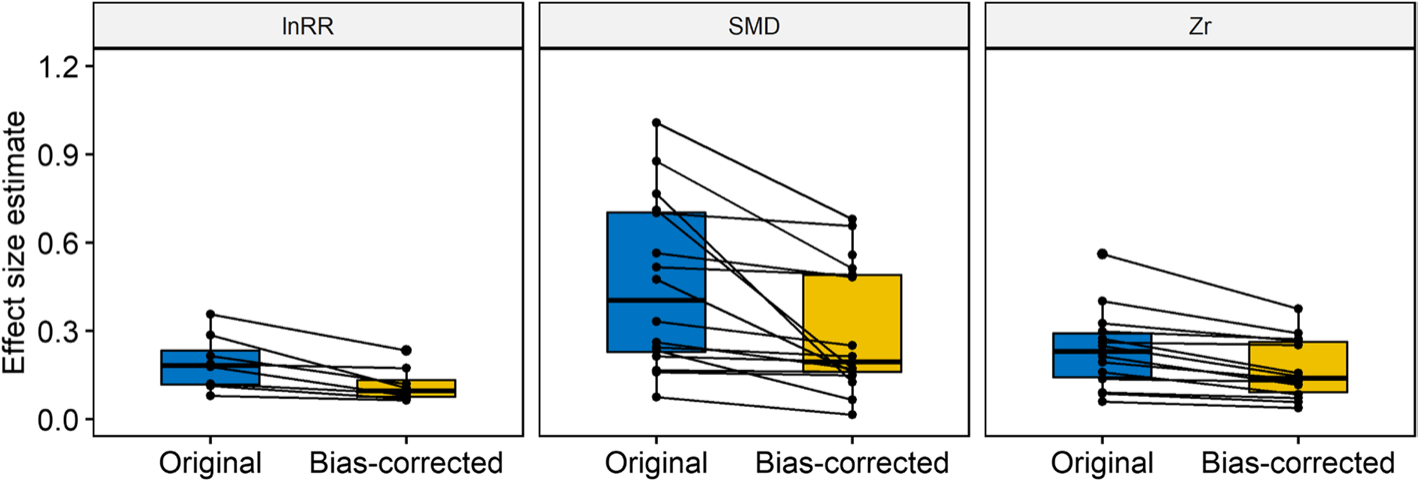
The magnitude of each meta-analysis' estimated effect size declines after correcting for publication bias. Nine of 20 meta-analyses of lnRR, 17 of 36 meta-analyses of SMD and 14 of 31 meta-analyses of *Zr* had corrected directions of slope after adjusting for publication bias. The remaining 11 in lnRR, 19 in SMD, and 17 in *Zr* had the wrong direction of slope, presumably because of a high degree of heterogeneity that could not be controlled for. Original = uncorrected meta-analytic estimate effect sizes (i.e. *β*_o[overall]_ in Equation 1). Bias-corrected = meta-analytic estimate effect size corrected for the presence of two forms of publication bias, small-study and decline effects (i.e. *β*_0[bias − corrected]_ in Equation 3)

Among the 87 meta-analyses examined, the estimated absolute mean difference between the original (uncorrected) effect size (*β*_0[overall]_) and its bias-corrected version (*β*_0[bias − corrected]_) was statistically significant (pooled *D* = 0.225, 95% CI = 0.180 to 0.269, *p*-value < 0.001; Fig. S1A). An overestimation of 0.189, 0.195 and 0.333 standard deviation units were found in SMD, lnRR, and *Zr*, respectively (Fig. S1B). After back-transformation to the original scale, the publication bias led to an exaggeration of the estimates of SMD, lnRR, and *Zr* by an average of 0.217, 0.116 and 0.128 (Fig. 7), respectively. Additionally, after correcting for publication bias, 33 out of 50 initially statistically significant meta-analytic means became non-significant.

### Statistical power and type S and M error rates

#### Sampling level (primary studies)

**Fig. 8.**
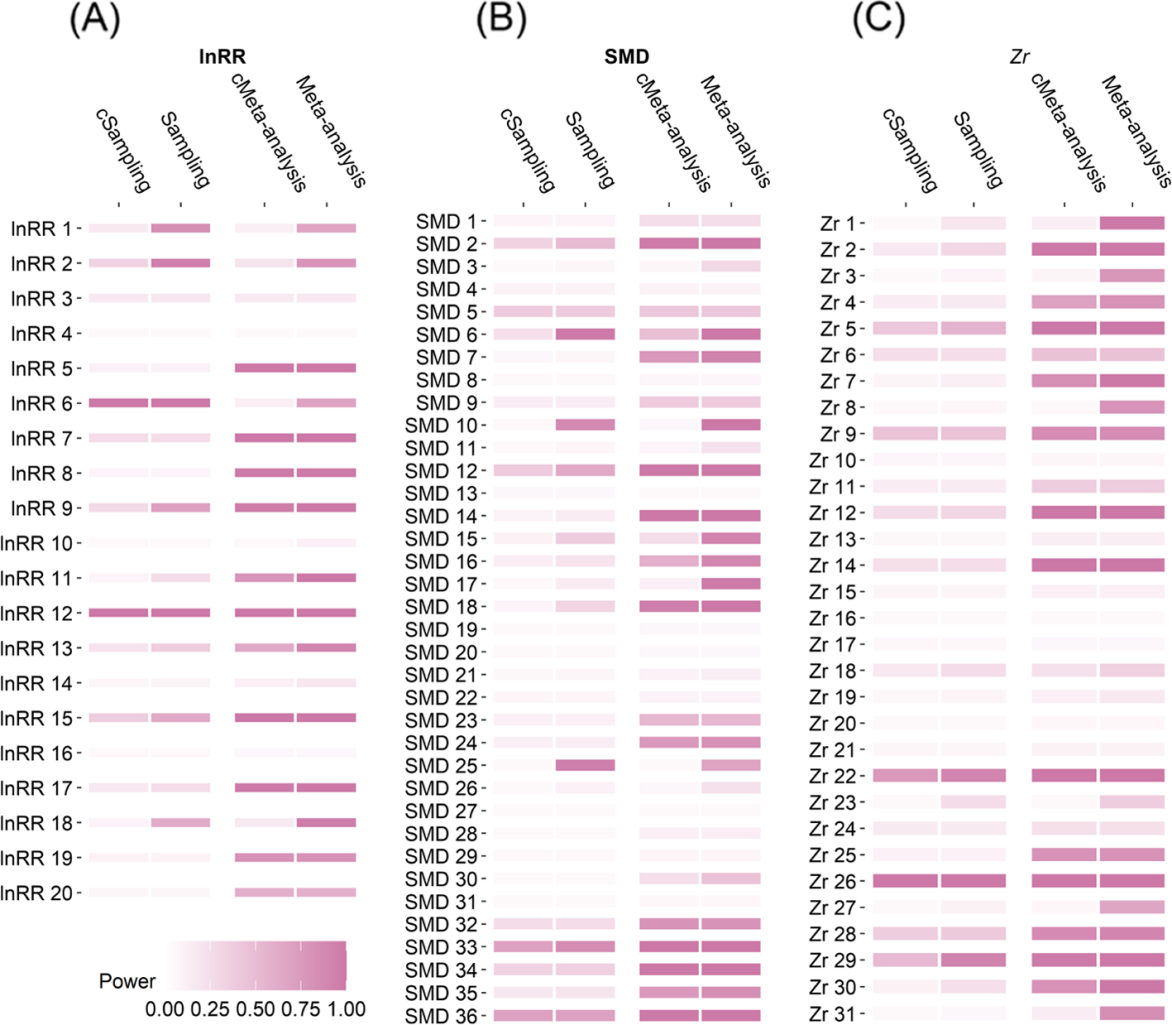
Ecological and evolutionary studies’ median statistical power to detect ‘true’ effects that were approximated by meta-analytic mean effect size estimates (labels: Meta-analysis, Sampling) and their bias-corrected versions (labels: cMeta-analysis, cSampling). On the y-axis, effect size metrics with different subscripts represent different individual meta-analyses (see Fig. 2). Sampling = statistical power at sampling level (primary studies). cSampling = statistical power at sampling level after correcting for publication bias. Meta-analysis = statistical power at meta-analysis level. cMeta-analysis = statistical power at meta-analysis level after correcting for publication bias. See more details in the legend of Fig. 3. All figures were drawn via *geom_tile()* function in *ggplot2* R package (version 2.0) [61]

Overall, primary studies or single experiments (i.e. at the sampling level) had a low statistical power of only 23% to detect the ‘true’ effect, as indicated by the original (uncorrected) meta-analytic estimate of effect sizes, *β*_0[overall]_. This was found to be the case across the different types of effect sizes, with power of 19%, 24% and 28% for sampling level of SMD, lnRR, and *Zr*, respectively (see Fig. 8 and Table S1). When bias correction was applied, the overall power to detect the ‘true’ effect (*β*_0[bias − corrected]_) decreased further to 15% (12%, 16%, and 18% for sampling level of SMD, lnRR, and *Zr*, respectively; see Fig. 8A and Table S1).

**Fig. 9.**
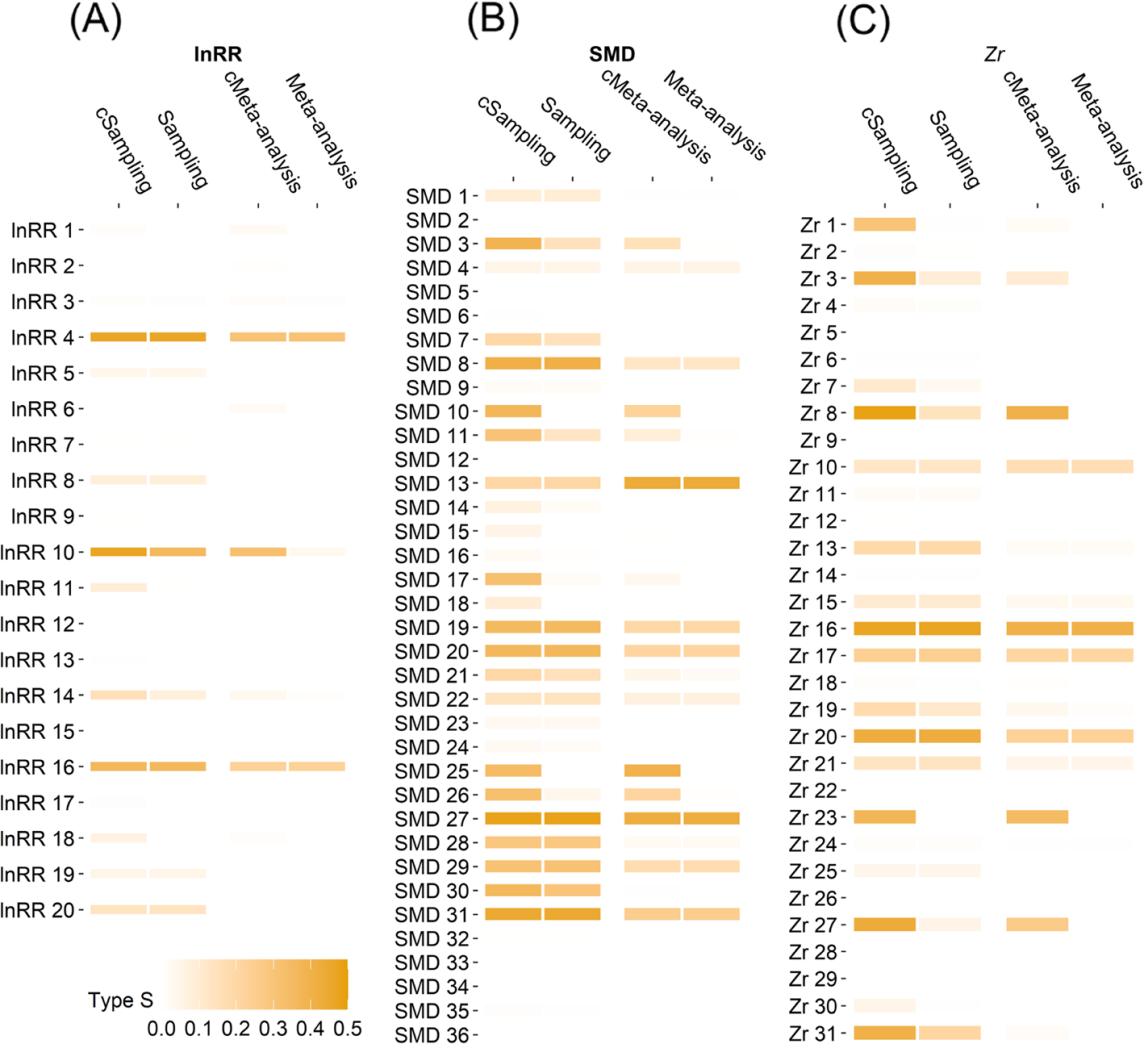
Ecological and evolutionary studies’ median Type S error rates (sign error) in detecting ‘true’ effects that were approximated by meta-analytic mean effect size estimates (labels: Meta-analysis, Sampling) and their bias-corrected versions (labels: cMeta-analysis, cSampling). On the y-axis, effect size metrics with different subscripts represent different individual meta-analyses (see Fig. 2). Sampling = statistical power at sampling level (primary studies). See more details in the legend of Figs. 3 and 8. All figures were drawn via *geom_tile()* function in *ggplot2* R package (version 2.0) [61]

The primary studies infrequently showed incorrect estimation of the signs of the true effect sizes (overall Type S error = 5%; Fig. 9 and Table S2). For example, the primary studies (i.e. at sampling level) using lnRR and SMD had only 5% and 6% probabilities of having a direction that was opposite to the meta-analytic mean estimated as *β*_0[overall]_. When correcting for publication bias the Type S error increased from 5% to 8%.

**Fig. 10.**
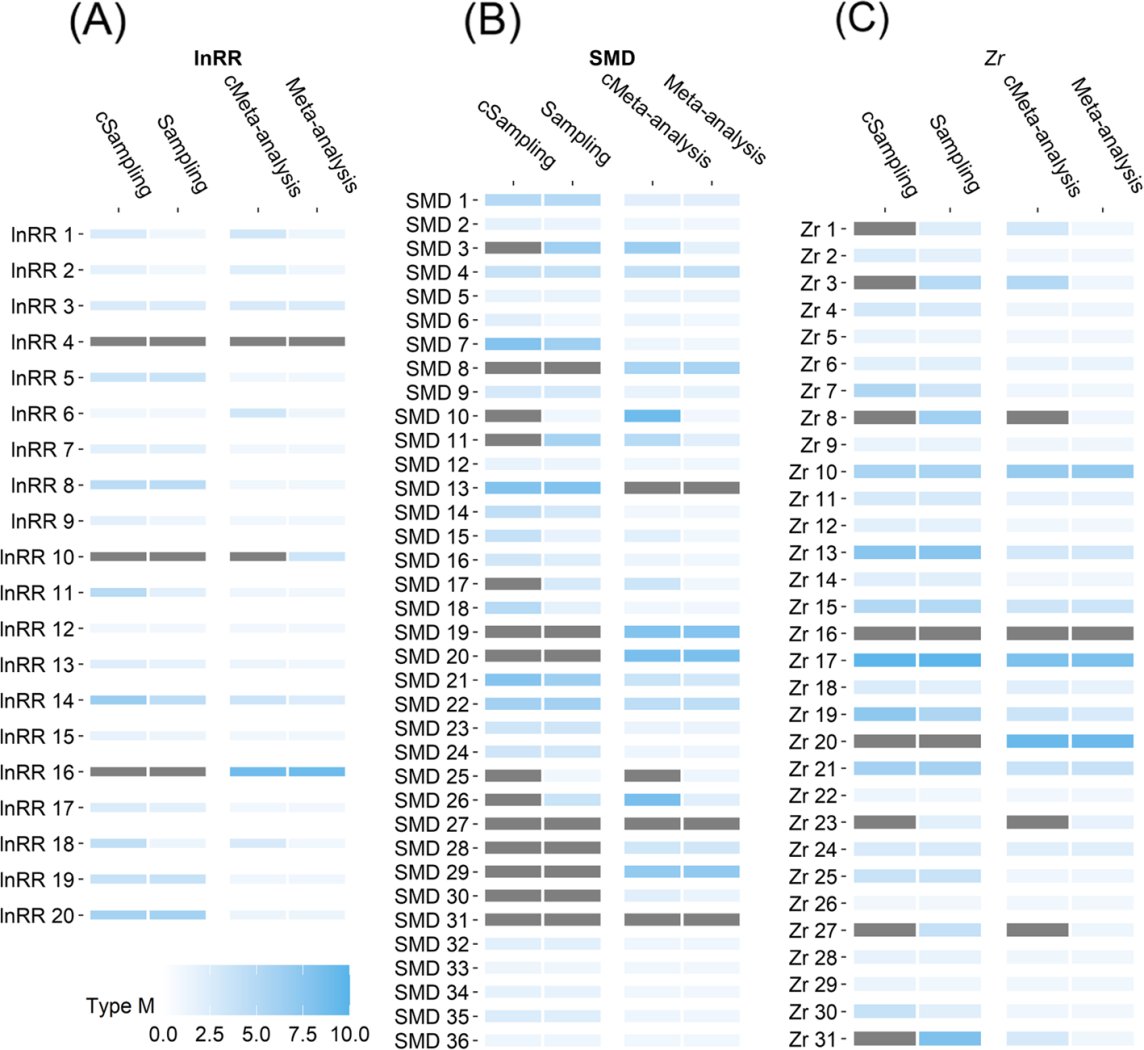
Ecological and evolutionary studies’ median Type M error rates (magnitude error) in detecting ‘true’ effects that were approximated by meta-analytic mean effect size estimates (labels: Meta-analysis, Sampling) and their bias-corrected versions (labels: cMeta-analysis, cSampling). On the y-axis, effect size metrics with different subscripts represent different individual meta-analyses (see Fig. 2). Grey cells indicate that Type M errors are greater than 10. See more details in the legend of Figs. 3 and 8. All figures were drawn via *geom_tile()* function in *ggplot2* R package (version 2.0) [61]

By contrast, the primary studies tended to exaggerate the magnitude of the meta-analytic mean estimated as *β*_0[overall]_, due to the limitation of finite sample size (overall Type M error = 2.7; Fig. 10 and Table S3). For example, the magnitude of lnRR, SMD and *Zr* were overestimated by an average of 2.5, 3.5 and 2 times, respectively. When correcting for publication bias (*β*_0[bias − corrected]_), the Type M errors increased to 4 (3.5 for lnRR, 6 for SMD and 3.4 for *Zr*).

#### Meta-analysis level

On average, at the level of individual meta-analyses, lnRR and *Zr* had statistical power that was at or above the nominal 80% level for detecting the true effects estimated as *β*_0[bias − corrected]_. Specifically, the power was found to be 81% for both lnRR and *Zr* (Fig. 8 and Table S1). In contrast, the estimated power of SMD was only 41%, which falls short of the nominal 80% level. When detecting true effects indicated by *β*_0[bias − corrected]_, the statistical power of each meta-analysis decreased further, with lnRR, SMD, and *Zr* decreasing to 63%, 25% and 51%, respectively.

Ecological and evolutionary meta-analyses had a relatively low probability of reporting an opposite sign to the true direction of both *β*_0[overall]_ and *β*_0[bias − corrected]_ (Type S = 5%–8%; Fig. 9 and Table S2). The meta-analyses were also able to considerably reduce the overestimation of the true effect size for lnRR (Type M = 1.1 for *β*_0[overall]_ and 1.3 for *β*_0[bias − corrected]_; Fig. 10 and Table S3), SMD (Type M = 1.9 for *β*_0[overall]_ and 2.5 for *β*_0[bias − corrected]_) and *Zr* (Type M = 1.1 for *β*_0[overall]_ and 1.6 for *β*_0[bias − corrected]_).

## Discussion

We have conducted the first comprehensive investigation of the prevalence and severity of two common forms of publication bias, small-study and decline effects) in the fields of ecology and evolutionary biology using modern analytic techniques. Overall, we found strong support for small-study and decline effects (time-lag bias) with little heterogeneity across studies. The prevalence of such publication bias resulted in overestimating meta-analytic mean effect size estimates by at least 0.12 standard deviations and substantially distorted the ecological and evolutionary evidence. The statistical power of ecological and evolutionary studies and experiments was found to be consistently low at 15%. Ecological and evolutionary studies also showed a 4-fold overestimation of effects (Type M error = 4.4) and a low but nontrivial rate of misidentifying the sign of the effects (Type S error = 8%; error in the direction that leads to the opposite conclusion). To place these in perspective with the replication crisis [5, 6], we conclude that prior published findings in ecology and evolutionary biology, at least for the dataset used in this study (87 meta-analyses, 4250 primary studies, 17,638 effect sizes) are likely to have low replicability.

### The persistent and non-negligible publication bias in ecological and evolutionary meta-analyses

#### Small-study and decline effects are general phenomena

We have found that 17% of ecological and evolutionary meta-analyses show evidence for small-study effects (i.e. smaller studies reporting larger effect sizes). Medical researchers found a similar percentage of meta-analyses showing small-study effects (7–18%) in a survey of 6873 meta-analyses (the large sample is because medical research has a bigger pool of meta-analyses to draw from and because that study extracted a much narrower scope of data from each meta-analysis than did our study [7, 63]). Similarly, 13–25% of psychological meta-analyses presented evidence for small-study effects [64, 65]). These values may seem relatively small, but this is in part because, for a given meta-analysis, bias detection methods often lack sufficient statistical power to identify a small-study effect [45, 63, 66]. Indeed, simulations have shown that the power to detect a moderate small-study effect in a medical meta-analysis with 10 studies was as low as 21% [14].

Given the limited power to detect a small-study effect [14], it seems reasonable to focus on the sign and magnitude of the relationship between effect size and sampling error rather than on *p*-values (i.e. null-hypothesis significance testing). By doing so, we found that more than 60% of meta-analyses had a positive statistically non-significant relationship between the effect size and its sampling error, indicating that small studies (i.e. with large sampling error or small precision) tend to report larger effects (note that the likelihood of meta-analysis showing this tendency is 50% under the null hypothesis). We confirmed these results by employing a more powerful approach, i.e. a second-order meta-analysis or meta-meta-analysis, which showed a statistically significant positive estimate of the relationship between effect size and sampling error. This result is in line with recent investigations revealing an negative mean association of effect size and sample size in psychology and psychiatry meta-analyses [51, 67]. Moreover, our analysis also showed a small amount of heterogeneity among these 87 slopes. This positive and homogenous effect implies that small-study effects are commonplace in ecology and evolutionary biology. Similar conclusions were reached in investigations of economic and psychological meta-analyses: small-study effects are widespread phenomena [68–70].

We conclude that decline effects are also widespread in the field. More than 50% of ecological and evolutionary meta-analyses showed a negative relationship between effect size and their year of publication, indicating that effect sizes decrease over time. As mentioned above, the principal reason for failing to detect a decline effect in a single meta-analysis lies in the low statistical power of the available detection methods [13, 45, 71]. The observed power to determine a decline effect in the current set of 87 meta-analyses was low (median = 13%), which is similar to that observed in another much larger survey of 464 ecological meta-analyses (median = 17%; [71, 72]). Importantly, our second-order meta-analysis found a statistically significant and homogeneous effect (Fig. 6A), corroborating that decline effects are common in both sub-fields previously explored (status signalling [73], plant and insect biodiversity [20, 74] and ocean acidification [75]) and more generally in ecology and evolutionary biology [12, 71]. Evidence from other disciplines also reveals the pervasiveness of decline effects (medical and social sciences [51, 76, 77]).

#### The distorted meta-analytic estimate of effect sizes and evidence by publication bias

By combining the observed bias from both small-study and decline effects, we found evidence that magnitudes of effect sizes might have been overestimated by 0.217, 0.116 and 0.128 SDs of their original units for lnRR, SMD and *Zr*, respectively. A recent investigation of 433 psychological meta-analyses also showed a statistically significant, albeit small, decrease in meta-analytic estimates after correcting for publication bias [78]. A comparison of meta-analyses that were published without pre-registration versus registered reports (which are less prone to publication bias) has also shown that unregistered meta-analyses substantially overestimated effect sizes although bias-correction methods like the one used in this study can correct for difference in results between meta-analyses and registered reports [79]. In our dataset, correcting for publication bias led to 33 of 50 initially statistically significant meta-analytic estimates of the mean effect becoming non-significant, suggesting unmerited confidence in the outcomes of 66% of published ecological and evolutionary meta-analyses (when using a frequentist approach with a *p*-value of 0.05). Recent psychological investigations revealed a similar percentage (60%) of erroneous conclusions of meta-analytic evidence because of publication bias [80].

### Low statistical power and high type M error in ecological and evolutionary studies

#### Ecological and evolutionary studies lack power and are prone to type M error

Primary studies in ecology and evolutionary biology included in our sample of meta-analyses, on average, only had a power of 15% to detect the biased-corrected effect size identified in the meta-analysis, which is consistent with earlier findings in the sub-fields of global change biology [56, 81] and animal behaviour [10, 23]. When excluding studies with effects that are not statistically significant, the corresponding average power of primary studies was still very low (17%; Table S4). As a result, only studies with largely exaggerated effect sizes (4-fold) have reached statistical significance. Contrastingly, Type S error was small but not trivial (8%); note that making an error in direction can result in a completely opposite conclusion. A lack of statistical power seems to be a general phenomenon in scientific research, low power has been identified in many disciplines (medical sciences = 20% [82], neuroscience = 21% [16], psychological sciences = 36% [27], economics = 18% [83]). Given this, meta-analysis with appropriate bias correction is an important way to generate more reliable estimates of effect sizes [30]. Statistically speaking, meta-analysis is an effective way to approximate population-level estimates by combining sampling level estimates, despite its shortcomings, some of which were shown above. Science is a process of evidence accumulation in which primary studies are the basis that can be used to produce high-order and high-quality evidence (e.g. via systematic review and meta-analysis).

#### Publication bias aggravates the low power and high Type M error

Publication bias is expected to reveal lower power and higher Type M error rates because it creates a non-random sample of effect size evidence used in meta-analyses. We show that correcting for publication bias resulted in a decrease in statistical power from 23% to 15%, an increase in Type S error rate from 5% to 8%, and an increase Type M error rates from 2.7 to 4.4. Psychological and economic research also confirm that meta-analyses without bias adjustments overestimate the estimate of statistical power [27, 28]. The exaggeration of power and effect size is even more severe in ecological and evolutionary studies if no bias correction is made [5], providing further support for recent concerns about the likelihood of low replicability (‘the replication crisis’) in ecology and evolutionary biology [6, 10].

### Limitations

There are four limitations in the present registered report. First, when calculating statistical power to detect true effects in ecology and evolutionary studies, we used the meta-analytic mean effect size (and corresponding bias-corrected version) as the true effect for each primary study within the same meta-analysis. This means that we assumed that the multiple primary studies included in the same meta-analysis share a common true effect. However, the high heterogeneity in ecology and evolutionary meta-analyses indicates that each primary study may have a specific true effect size that is dependent on the research context (e.g. population, species, methodology, lab effects [47]). Therefore, using such context-dependent effects as the proxies of true effect is probably more reasonable [81]. Second, in the post hoc analysis, we used the statistical significance (*p*-value < 0.05) of the meta-analytic mean effect size as the threshold to decide whether the true effect in a meta-analysis is so tiny that can be biologically neglected and subsequently excluded to calculate average power. We acknowledge that this categorisation is arbitrary because the statistical significance does not represent biological significance [4]. In some fields, very small effects still have biological importance. Third, the meta-analytic effect size estimates after correcting for publication bias may still be overestimated or underestimated because the incomplete reporting of important moderators in meta-analyses prevented us from accurately correcting for publication bias using our regression-based method [42, 46]. Fourth, notably, in testing for publication bias at both the within- and between-meta-analysis levels, we used statistical significance at the 0.05 level as a criteria to determine if there was publication bias. We acknowledge that this process, which is commonly referred to as a "significance filter", is prone to exaggeration and might result in a so-called "winner's curse" [84–86]. To partially mitigate this issue, the percentage of both statistically significant and non-significant results was reported in Figs. 4, 5 and 6. Furthermore, to avoid drawing conclusions based solely on statistically significant results, downstream analyses were conducted to assess the extent to which publication bias distorted the estimates of effect size (as shown in Fig. 7) and the calculation of power and Type M/S error rates (as shown in Figs. 8, 9 and 10).

### Implications

#### How to properly test for publication bias and correct for its impacts?

Given the strong and widespread evidence of publication bias found in this study (and others), publication bias tests should be a standard part of meta-analyses. A recent survey showed that publication bias tests have become more widespread in ecology and evolution in recent years [45]; however, inappropriate bias detection methods still dominate the literature [45]. Generally, regression-based methods are more powerful than other methods such as correlation-based methods [14, 63]. The regression-based method in the multilevel model framework used in the current study can further handle non-independence and high heterogeneity, which are common in the field, to bring down the rate of false positives [45–47]. Importantly, the method used here provides an intuitive quantification of the severity of publication bias. For example, the pooled *β*_1[small − study]_ of *Zr* was larger than that of SMD (0.119 *vs.* 0.091), suggesting publication bias in *Zr* is more severe than in SMD. Regression-based methods to correct for publication bias have been shown to produce effect size estimates similar to those of registered reports [79]. We strongly recommend that meta-analysts employ the regression-based method used in the current paper to routinely test for the presence of publication bias, correct for its impact and, report the corrected effect sizes, allowing stakeholders to better judge how robust the reported effects are.

#### How to increase power and mitigate overestimation of effect for primary studies and meta-analyses?

For primary studies, a fundamental solution to increase statistical power and mitigate effect size overestimation is to increase sample sizes by building up more big-team science [87] or global-scale collaborative scientific networks such as Nutrient Network [88], US Long-Term Ecological Research network [89], and Zostera Experimental Network [90]. Our results confirm that lnRR is a more powerful effect size metric than SMD [81]. Power of meta-analyses using lnRR was almost twice as large as SMD (lnRR *vs.* SMD: 81% *vs.* 41%). Moreover, lnRR was less prone to exaggeration (lnRR *vs.* SMD: 1 *vs.* 2). Practically, we recommend using lnRR as the main effect size when conducting meta-analyses if the biological questions focused on mean differences (but see [91]), but conduct sensitivity analyses using SMD (see [81, 92] for comparisons of the pros and cons of lnRR and SMD).

## Conclusions

We indirectly examined the extent of the replication crisis in ecology and evolutionary biology using two inter-related indicators: publication bias and statistical power. Our results indicate that two expected outcomes of publication bias, small-study effects and decline effects, are persistent and non-negligible in the field. Primary studies in ecology and evolutionary biology are often underpowered and prone to overestimate the magnitude of the effect (i.e. Type M error). Pervasive publication bias leads to exaggerated effect sizes, inflated meta-analytic evidence and overestimated statistical power, and to underestimated Type M error rates, undermining the reliability of previous findings. Although no single indicator can capture the true extent or all relevant evidence of the replication crisis [93], we have provided clear evidence that, as in many other disciplines [1, 2, 4], previously published findings in ecology and evolutionary biology are likely to have low replicability. The likely replication crisis in these fields highlights the importance of (i) designing high-power primary studies by building up big-team science [7, 87] where possible, (ii) adopting appropriate publication bias detection and correction methods for meta-analyses [45], (iii) embracing publication-bias-robust publication forms (e.g. Registered Reports — like the current article) for both empirical studies and meta-analyses alike. More generally, researchers need to adhere more closely to open and transparent research practices [94], such as (pre-)registration [95], data and code sharing [96, 97], and transparent reporting [5], to achieve credible, reliable and reproducible ecology and evolutionary biology.

## Supplementary Information

**Additional file 1.** Supporting Information.

## Data Availability

The relevant data and code that reproduce the results of this registered report are available at GitHub Repository (https://github.com/Yefeng0920/EcoEvo_PB) and Zenodo (Yefeng0920/EcoEvo_PB: Registered Report - publicaiton bias in Eco & Evo. DOI: 10.5281/zenodo.7762126).
