# Supplementary material for: Publication bias impacts on effect size, statistical power, and magnitude (Type M) and sign (Type S) errors in ecology and evolutionary biology
Source: BMC Biol. 2023 Apr 3;21:71. doi: 10.1186/s12915-022-01485-y (PMC10071700; doi:10.1186/s12915-022-01485-y)
Supplement: Supplementary file 1 — Additional file 1. Supporting Information. [file 12915_2022_1485_MOESM1_ESM.docx]

**Supporting Information**

Publication bias impacts on effect size, statistical power, and magnitude (Type M) and sign (Type S) errors in ecology and evolutionary biology

Yefeng Yang^1,2,3*^, Alfredo Sánchez-Tójar^4^, Rose E. O’Dea^5^, Daniel W.A. Noble^6^, Julia Koricheva^7^, Michael D. Jennions^6^, Timothy H. Parker^8^, Malgorzata Lagisz^1,9^, Shinichi Nakagawa^1,9*^

^1^ Evolution & Ecology Research Centre and School of Biological, Earth and Environmental Sciences, University of New South Wales, Sydney, NSW 2052, Australia

^2^ Department of Biosystems Engineering, Zhejiang University, Hangzhou 310058, China

^3^ Department of Infectious Diseases and Public Health, Jockey Club College of Veterinary Medicine and Life Sciences, City University of Hong Kong, Hong Kong, China

^4^ Department of Evolutionary Biology, Bielefeld University, Bielefeld, 33615, Germany

^5^ School of Ecosystem and Forest Sciences, University of Melbourne, Parkville, Australia

^6^ Division of Ecology and Evolution, Research School of Biology, The Australian National University, Canberra, ACT, Australia

^7^ Department of Biological Sciences, Royal Holloway University of London, Egham, Surrey, TW20 0EX, U.K.

^8^ Department of Biology, Whitman College, Walla Walla, WA 99362, U.S.A

^9^ Equal contribution as senior authors.

*Correspondence: Y. Yang, e-mail: [yefeng.yang1@unsw.edu.au](mailto:yefeng.yang1@unsw.edu.au); S. Nakagawa, e-mail: [s.nakagawa@unsw.edu.au](mailto:s.nakagawa@unsw.edu.au)

**ORCID**

Yefeng Yang: 0000-0002-8610-4016; Alfredo Sánchez-Tójar: 0000-0002-2886-0649; Rose E. O’Dea: 0000-0001-8177-5075; Daniel W. A. Noble: 0000-0001-9460-8743; Julia Koricheva: 0000-0002-9033-0171; Michael D. Jennions: 0000-0001-9221-2788; Timothy H. Parker: 0000-0003-2995-5284; Malgorzata Lagisz: 0000-0002-3993-6127; Shinichi Nakagawa: 0000-0002-7765-5182


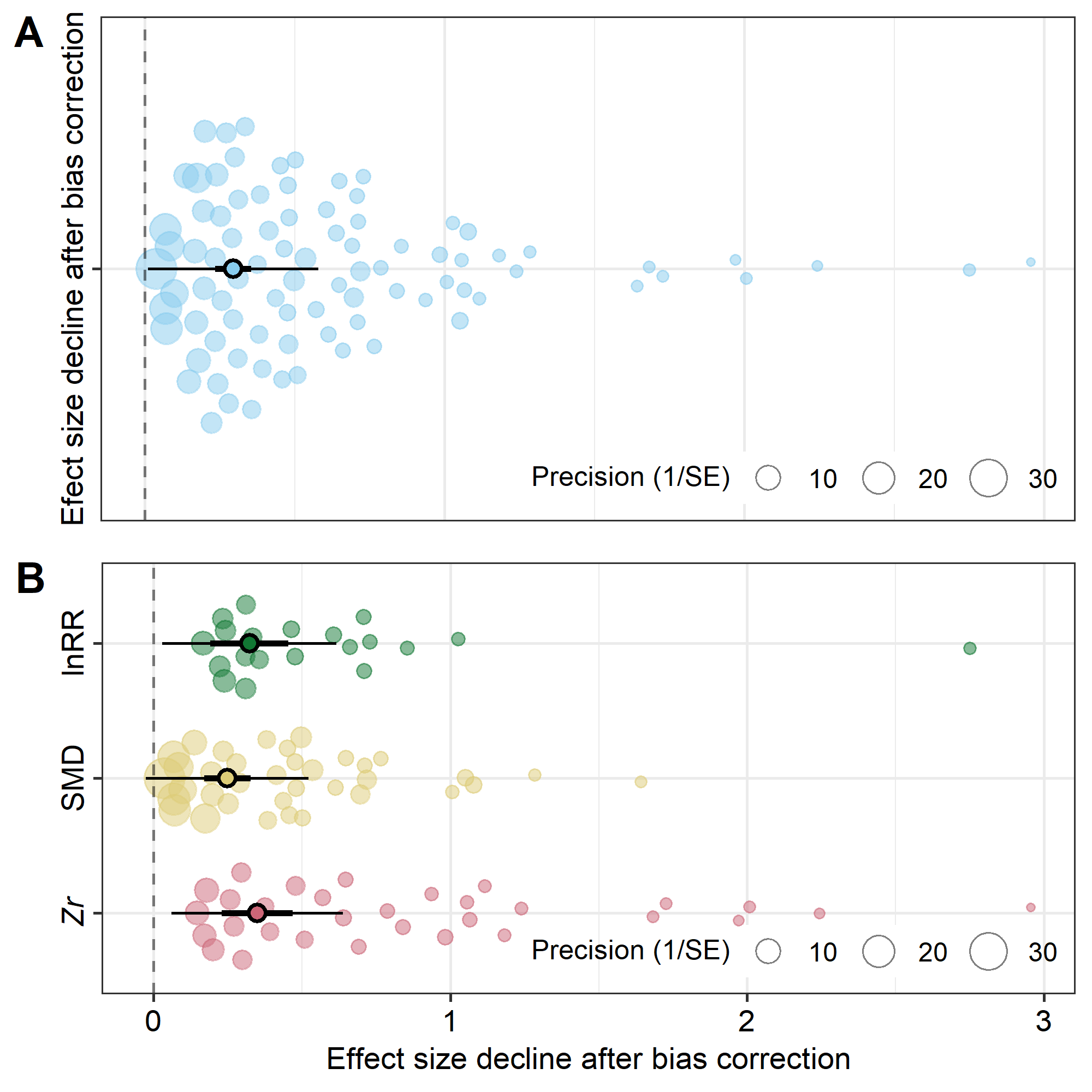


**Figure S1**

The reduction in effect-size magnitude after controlling for publication bias. We used the absolute mean difference (D) as a metric to quantify the reduction in effect-size magnitude following correction for publication bias. (A) Pooled D across different meta-analyses (B) Pooled D for each type of effect size. Solid circles = D statistics obtained from each meta-analysis; the size of each solid circle is proportional to its inverse standard error (i.e., precision). Open circles = pooled D statistic. Thick error bars = 95% confidence intervals (CIs). Thin error bars = prediction intervals (PIs). All panels were made using *orchard_plot()* function in *orchaRd* R package (version 2.0) [1]. The formula to calculate D statistic for each meta-analysis can be found at Equation 9 in the main text. Note that D statistic is an absolute variable that follows a ‘folded’ normal distribution because taking the absolute value will force probability density on its left side (x-axis < 0) to be folded to the right. Therefore, we used the technique of meta-analysis of magnitude to statistically aggregate the D obtained from each meta-analysis.

**Table S1**

The model estimates of statistical power to detect true effects that were approximated by uncorrected ($\beta_{o[overall]}$) and bias-corrected effect sizes ($\beta_{0[bias-corrected]}$). The model estimates of power were reported both on sampling/experimental level and meta-analysis level. We used mixed-effects models and weighted regression models to average over sampling/experimental level statistical power and meta-analysis level statistical power, respectively. Note that: (1) the confidence intervals (CI) of statistical estimate were asymmetrical due to the back-transformation, (2) statistical power estimates below or above the boundary values (i.e., 0 or 1) were constrained to the boundaries (i.e., 0^#^ or 1^#^). $\beta_{o[overall]}$ = (original) uncorrected meta-analytic estimate effect sizes (see details in Equation 2), $\beta_{0[bias-corrected]}$ = meta-analytic estimate effect size corrected for the presence of two forms of publication bias, small-study and decline effects, *k* = the number of effect sizes, *N* = the number of primary studies.

|  | Effect size types | True effects | Statistical power | | | *k* | *N* |
| --- | --- | --- | --- | --- | --- | --- | --- |
|  |  |  | Median | CI.lb | CI.ub |  |  |
| Sampling level |  |  |  |  |  |  |  |
|  | All | $\beta_{o[overall]}$ | 0.23 | 0.22 | 0.24 | 17638 | 4250 |
|  |  | $\beta_{0[bias-corrected]}$ | 0.15 | 0.14 | 0.15 | 17638 | 4250 |
|  |  |  |  |  |  |  |  |
|  | SMD | $\beta_{o[overall]}$ | 0.19 | 0.18 | 0.20 | 17638 | 4250 |
|  |  | $\beta_{0[bias-corrected]}$ | 0.12 | 0.11 | 0.13 | 17638 | 4250 |
|  |  |  |  |  |  |  |  |
|  | lnRR | $\beta_{o[overall]}$ | 0.24 | 0.22 | 0.25 | 17638 | 4250 |
|  |  | $\beta_{0[bias-corrected]}$ | 0.16 | 0.15 | 0.17 | 17638 | 4250 |
|  |  |  |  |  |  |  |  |
|  | *Zr* | $\beta_{o[overall]}$ | 0.28 | 0.27 | 0.30 | 17638 | 4250 |
|  |  | $\beta_{0[bias-corrected]}$ | 0.19 | 0.18 | 0.20 | 17638 | 4250 |
|  |  |  |  |  |  |  |  |
| Meta-analysis |  |  |  |  |  |  |  |
|  | All | $\beta_{o[overall]}$ | 0.55 | 0.45 | 0.68 | 17638 | 4250 |
|  |  | $\beta_{0[bias-corrected]}$ | 0.36 | 0.28 | 0.45 | 17638 | 4250 |
|  |  |  |  |  |  |  |  |
|  | SMD | $\beta_{o[overall]}$ | 0.41 | 0.28 | 0.6 | 17638 | 4250 |
|  |  | $\beta_{0[bias-corrected]}$ | 0.25 | 0.17 | 0.36 | 17638 | 4250 |
|  |  |  |  |  |  |  |  |
|  | lnRR | $\beta_{o[overall]}$ | 0.81 | 0.64 | 1# | 17638 | 4250 |
|  |  | $\beta_{0[bias-corrected]}$ | 0.63 | 0.43 | 0.91 |  |  |
|  |  |  |  |  |  |  |  |
|  | *Zr* | $\beta_{o[overall]}$ | 0.81 | 0.65 | 1# | 17638 | 4250 |
|  |  | $\beta_{0[bias-corrected]}$ | 0.51 | 0.35 | 0.75 | 17638 | 4250 |

**Table S2**

The model estimates of Type S error rates in detecting true effects that were approximated by uncorrected ($\beta_{o[overall]}$) and bias-corrected effect sizes ($\beta_{0[bias-corrected]}$). The model estimates of Type S errors were reported both on sampling/experimental level and meta-analysis level. Details see Table S1

|  | Effect size types | True effects | Type S error rates | | | *k* | *N* |
| --- | --- | --- | --- | --- | --- | --- | --- |
|  |  |  | Median | CI.lb | CI.ub |  |  |
| Sampling level |  |  |  |  |  |  |  |
|  | All | $\beta_{o[overall]}$ | 0.05 | 0.05 | 0.05 | 17638 | 4250 |
|  |  | $\beta_{0[bias-corrected]}$ | 0.08 | 0.08 | 0.08 | 17638 | 4250 |
|  |  |  |  |  |  |  |  |
|  | SMD | $\beta_{o[overall]}$ | 0.06 | 0.06 | 0.07 | 17638 | 4250 |
|  |  | $\beta_{0[bias-corrected]}$ | 0.11 | 0.10 | 0.11 | 17638 | 4250 |
|  |  |  |  |  |  |  |  |
|  | lnRR | $\beta_{o[overall]}$ | 0.05 | 0.05 | 0.05 | 17638 | 4250 |
|  |  | $\beta_{0[bias-corrected]}$ | 0.07 | 0.07 | 0.07 | 17638 | 4250 |
|  |  |  |  |  |  |  |  |
|  | *Zr* | $\beta_{o[overall]}$ | 0.04 | 0.04 | 0.04 | 17638 | 4250 |
|  |  | $\beta_{0[bias-corrected]}$ | 0.06 | 0.07 | 0.07 | 17638 | 4250 |
|  |  |  |  |  |  |  |  |
| Meta-analysis |  |  |  |  |  |  |  |
|  | All | $\beta_{o[overall]}$ | 0.03 | 0.03 | 0.04 | 17638 | 4250 |
|  |  | $\beta_{0[bias-corrected]}$ | 0.04 | 0.03 | 0.05 | 17638 | 4250 |
|  |  |  |  |  |  |  |  |
|  | SMD | $\beta_{o[overall]}$ | 0.04 | 0.03 | 0.06 | 17638 | 4250 |
|  |  | $\beta_{0[bias-corrected]}$ | 0.05 | 0.04 | 0.07 | 17638 | 4250 |
|  |  |  |  |  |  |  |  |
|  | lnRR | $\beta_{o[overall]}$ | 0.03 | 0.02 | 0.03 | 17638 | 4250 |
|  |  | $\beta_{0[bias-corrected]}$ | 0.03 | 0.02 | 0.04 |  |  |
|  |  |  |  |  |  |  |  |
|  | *Zr* | $\beta_{o[overall]}$ | 0.03 | 0.02 | 0.03 | 17638 | 4250 |
|  |  | $\beta_{0[bias-corrected]}$ | 0.03 | 0.03 | 0.05 | 17638 | 4250 |

**Table S3**

The model estimates of Type M error rates in detecting true effects that were approximated by uncorrected ($\beta_{o[overall]}$) and bias-corrected effect sizes ($\beta_{0[bias-corrected]}$). The model estimates of Type M errors were reported both on sampling/experimental level and meta-analysis level. Details see Table S1

|  | Effect size types | True effects | Type M error rates | | | *k* | *N* |
| --- | --- | --- | --- | --- | --- | --- | --- |
|  |  |  | Median | CI.lb | CI.ub |  |  |
| Sampling level |  |  |  |  |  |  |  |
|  | All | $\beta_{o[overall]}$ | 2.73 |  |  | 17638 | 4250 |
|  |  | $\beta_{0[bias-corrected]}$ | 4.43 |  |  | 17638 | 4250 |
|  |  |  |  |  |  |  |  |
|  | SMD | $\beta_{o[overall]}$ | 3.48 |  |  | 17638 | 4250 |
|  |  | $\beta_{0[bias-corrected]}$ | 6.02 |  |  | 17638 | 4250 |
|  |  |  |  |  |  |  |  |
|  | lnRR | $\beta_{o[overall]}$ | 2.5 |  |  | 17638 | 4250 |
|  |  | $\beta_{0[bias-corrected]}$ | 3.47 |  |  | 17638 | 4250 |
|  |  |  |  |  |  |  |  |
|  | *Zr* | $\beta_{o[overall]}$ | 2.1 |  |  | 17638 | 4250 |
|  |  | $\beta_{0[bias-corrected]}$ | 3.43 |  |  | 17638 | 4250 |
|  |  |  |  |  |  |  |  |
| Meta-analysis |  |  |  |  |  |  |  |
|  | All | $\beta_{o[overall]}$ | 1.5 | 1.27 | 1.76 | 17638 | 4250 |
|  |  | $\beta_{0[bias-corrected]}$ | 1.97 | 1.64 | 2.37 | 17638 | 4250 |
|  |  |  |  |  |  |  |  |
|  | SMD | $\beta_{o[overall]}$ | 1.86 | 1.37 | 2.52 | 17638 | 4250 |
|  |  | $\beta_{0[bias-corrected]}$ | 2.54 | 1.86 | 3.46 | 17638 | 4250 |
|  |  |  |  |  |  |  |  |
|  | lnRR | $\beta_{o[overall]}$ | 1.13 | 0.95 | 1.34 | 17638 | 4250 |
|  |  | $\beta_{0[bias-corrected]}$ | 1.32 | 1.02 | 1.69 |  |  |
|  |  |  |  |  |  |  |  |
|  | *Zr* | $\beta_{o[overall]}$ | 1.14 | 0.97 | 1.34 | 17638 | 4250 |
|  |  | $\beta_{0[bias-corrected]}$ | 1.56 | 1.16 | 2.1 | 17638 | 4250 |

**Table S4**

The model estimates of statistical power to detect true effects that were categorized by the statistical significance of meta-analytic mean effect ($\beta_{o[overall]}$) and bias-corrected effect sizes ($\beta_{0[bias-corrected]}$). The model estimates of statistical power were reported both on sampling/experimental level and meta-analysis level. Details see Table S1

|  | p-value of $\beta_{o}$ | True effects | Statistical power | | | *k* | *N* |
| --- | --- | --- | --- | --- | --- | --- | --- |
|  |  |  | Median | CI.lb | CI.ub |  |  |
| Sampling level |  |  |  |  |  |  |  |
|  | < 0.05 | $\beta_{o[overall]}$ | 0.29 | 0.28 | 0.30 | 17638 | 4250 |
|  |  | $\beta_{0[bias-corrected]}$ | 0.17 | 0.17 | 0.18 | 17638 | 4250 |
|  |  |  |  |  |  |  |  |
|  | > 0.05 | $\beta_{o[overall]}$ | 0.08 | 0.07 | 0.08 | 17638 | 4250 |
|  |  | $\beta_{0[bias-corrected]}$ | 0.07 | 0.07 | 0.07 | 17638 | 4250 |
|  |  |  |  |  |  |  |  |
| Meta-analysis |  |  |  |  |  |  |  |
|  | < 0.05 | $\beta_{o[overall]}$ | 0.93 | 0.90 | 0.97 | 17638 | 4250 |
|  |  | $\beta_{0[bias-corrected]}$ | 0.57 | 0.45 | 0.72 | 17638 | 4250 |
|  |  |  |  |  |  |  |  |
|  | > 0.05 | $\beta_{o[overall]}$ | 0.12 | 0.09 | 0.16 | 17638 | 4250 |
|  |  | $\beta_{0[bias-corrected]}$ | 0.10 | 0.08 | 0.12 | 17638 | 4250 |

**References**

1. Nakagawa S, Lagisz M, O'Dea RE, Rutkowska J, Yang Y, Noble DW, Senior AM: **The orchard plot: Cultivating a forest plot for use in ecology, evolution, and beyond**. *Research Synthesis Methods* 2021, **12**(1):4-12.
